# Prospects for Development and Commercialisation of Allogeneic CAR-Based Therapies for Autoimmune Disease

**DOI:** 10.3390/biology14121790

**Published:** 2025-12-15

**Authors:** Madeleine Osborne, John Maher

**Affiliations:** 1 School of Medicine, King’s College London, 1st Floor, Henriette Raphael Building, Guy’s Hospital Campus Great Maze Pond, London SE1 1UL, UK; madeleine.osborne@kcl.ac.uk; 2 CAR Mechanics Group, Guy’s Cancer Centre, School of Cancer and Pharmaceutical Sciences, King’s College London, Great Maze Pond, London SE1 9RT, UK; 3 Leucid Bio Ltd., Guy’s Hospital, Great Maze Pond, London SE1 9RT, UK; 4 Department of Immunology, Eastbourne Hospital, Kings Drive, Eastbourne BN21 2UD, UK

**Keywords:** chimeric antigen receptor, allogeneic, autoimmune disease, CAR-T cell, off the shelf

## Abstract

Chimeric antigen receptor (CAR)-T cell therapies represent a promising therapeutic approach for refractory autoimmune diseases. Although autologous CAR-T cells have achieved success thus far, they require expensive, individualised manufacturing, limiting their commercialisation potential. Allogeneic alternatives could overcome these scalability barriers, providing ‘off-the-shelf’ treatments, although they raise the issues of graft-vs-host reactions and host-mediated rejection. To mitigate such risks, gene-edited αβ T cells or non-alloreactive host cells (e.g., NK cells, γδ T cells) may be used. This review evaluates evidence of the functionality and commercial potential of various allogeneic CAR-T solutions for autoimmunity. Searches were conducted of PubMed, EMBASE and Web of Science to extract clinical and preclinical studies of allogeneic CAR-T cells, for the treatment of autoimmune diseases and B or T cell malignancies. In light of the paucity of data on autoimmune disease, the latter were included to facilitate extrapolation to the autoimmune setting. A total of 107 studies were included. The available clinical outcomes of efficacy and safety, as well as preclinical key findings, are reported. Current developments and potential future improvements for safety, effectiveness and cost-effective manufacture are then discussed. The findings of this review demonstrate the promising therapeutic potential of allogeneic CAR-T for autoimmune disease, with scope for the further optimisation of safety and scalable manufacture to facilitate commercialisation.

## Simple Summary

Autoimmune diseases often progress to become resistant to currently available treatments. Recently, evidence has emerged to indicate that immune white blood cells, called T cells, from patients with severe autoimmune disease can be re-programmed in the laboratory by the introduction of a new gene. When infused back into patients, these so-called CAR-T cells can achieve dramatic remission of disease. Unfortunately, however, this is a very cumbersome and costly treatment option. This review examines alternative approaches to produce CAR-T cells using healthy donor blood as a means to reduce costs and enhance availability for patients, while maintaining effective control of autoimmune disease.

## 1. Introduction

### 1.1. Chimeric Antigen Receptors

**Figure 1:**
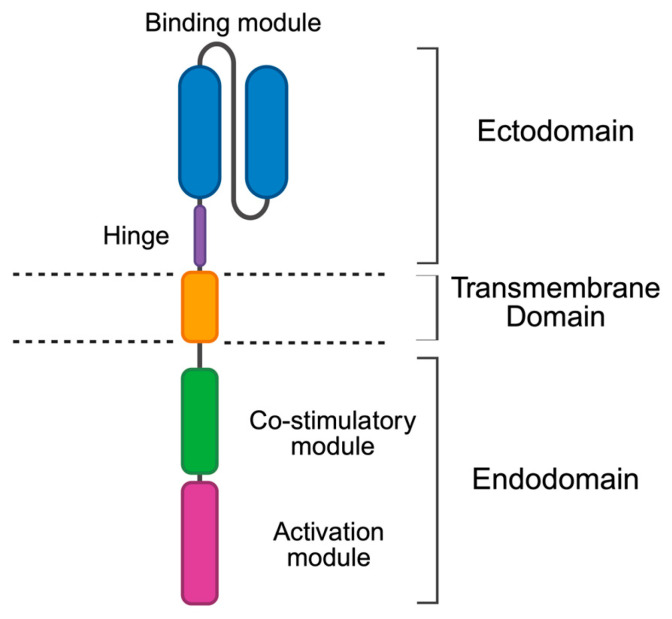
Cartoon illustration of the structure of a second-generation CAR. The composition of individual components that have been fused to generate this synthetic receptor is illustrated.

Chimeric antigen receptors (CARs) are synthetic fusion receptors in which one or more binding domains are fused via a spacer/hinge and transmembrane region to a bespoke signalling domain. They comprise four elements, namely a signalling endodomain, a transmembrane domain, a hinge/spacer domain and a ligand-binding ectodomain, most commonly a single-chain antibody fragment with specificity for a native cell surface antigen. This structure contrasts with a typical αβ T cell receptor (TCR), which recognises a processed antigen-derived peptide that is presented in the context of a Major Histocompatibility Complex (MHC) molecule. All approved CARs are of “second generation” design (illustrated schematically in Figure 1) in which a single co-stimulatory domain is placed upstream of an activating domain. When expressed in T cells, CARs redirect their specificity in an MHC-independent manner against target cells that express cognate native cell surface antigens.

Immunotherapy using CAR-T cells has achieved striking success in patients with selected haematological malignancies [1]. Currently, there are seven Food and Drug Administration (FDA)-approved CAR-T cell therapies for refractory blood cancers. The efficient elimination of malignant immune cells using CAR-T has ignited the idea that pathological immune cells could similarly be targeted to establish novel therapies for refractory autoimmune disease [2]. Recent trials have provided impressive clinical proofs of concept for this principle, with CD19-specific CAR-T products inducing complete clinical remission in multiple subjects with refractory Systemic Lupus Erythematosus (SLE) [3]. Subsequent reports have confirmed the durability of these responses for up to 4 years in SLE, inflammatory myositis and systemic sclerosis [4,5]. Notably, however, such trials have evaluated autologous CAR-T, manufactured from a patient’s own cells. This is a labour-intensive, lengthy and expensive process, creating a practical barrier to the commercialisation of such therapies [2]. Allogeneic CAR-T cells, sourced from healthy donors, could provide a cheaper, more scalable solution, generating ‘off-the-shelf’ therapies [6]. However, allogeneic donor-derived products can carry a substantial risk of both graft-vs.-host disease (GvHD) and host-mediated rejection [7], raising concerns over the efficacy and safety of this approach. One potential solution to both issues entails the use of genome editing to negate both the alloreactivity and immunogenicity of CAR-T products, through the elimination of αβ TCR and mismatched HLA molecule expression, respectively [7]. An alternative, non-genome-editing method involves the manufacture of CAR therapies from ‘unconventional’ T cell subsets instead of αβ T cells, such as Natural Killer (NK) or Gamma Delta (γδ) cells, that recognise target cells in a Major Histocompatibility Complex (MHC)-independent manner, mitigating the risk of GvHD. Furthermore, these two methods may be combined in order to enhance ‘non-genome-edited’, inherently cytotoxic CAR products, providing additional gene edits to prevent rejection, improve persistence and/or efficacy [8]. These approaches each have their own benefits and drawbacks with regard to function and manufacturing, but all share the potential to yield fully allogeneic CAR-T cell therapy. The objective of this review is to evaluate the development potential of allogeneic CAR-T therapies, using both genome-editing and non-genome-editing strategies, assessing progress thus far and looking to the future, including prospects for their commercialisation as an autoimmune disease therapeutic modality. Searches of these two technologies were conducted using PubMed, EMBASE (Ovid) and the Web of Science Core Collection in November 2024, March 2025 and November 2025 (see Supplementary Materials for details of methods). The review questions and eligibility criteria were defined using a PICO [9] format (Figure S1 and Figure S2, respectively). Search terms were combined using Boolean Operators (Figure S3). Given the relative lack of data pertaining to autoimmunity, searches also evaluated the use of allogeneic CAR-T for the treatment of B or T cell malignancies, to facilitate extrapolation to the autoimmune setting. In this context, we have not appraised other competing approaches, such as in vivo CAR-T or CAR-engineered regulatory T cells.

### 1.2. Autoimmune Disease and CAR-T

In autoimmune disease, the immune system mistakes healthy tissues as foreign and attacks them owing to autoreactive B and T cells [10]. B cells act as antigen-presenting cells and produce autoantibodies, while T cells cause direct tissue damage and produce inflammatory cytokines [2]. Increasingly, it is recognised that B cells have a fundamental role in the loss of immune tolerance, leading to autoimmune disease. Tolerance breaks down at multiple anatomic sites (bone marrow and spleen in mice, less certain in humans) and at distinct stages of B cell development/maturation, including V(D)J recombination in bone marrow and somatic mutation at germinal centre and extrafollicular sites [11]. As a result, more self-reactive B cells are retained in patients with autoimmune disease compared to healthy subjects. For this reason, B cell-targeted therapies have been in use for autoimmune disorders for many years. However, autologous B cell-targeted CAR T cells have achieved profound efficacy against refractory B cell disorders in preliminary studies, as summarised above. A key emerging concept is the fact that CAR T cells (unlike other therapies such as monoclonal antibodies) can achieve a deep depletion of both circulating and tissue-resident B cells, eliciting an ‘immune reset’ that leads to sustained disease remission [3]. In this context, the term immune reset refers to the profound depletion of B cells across the differentiation spectrum, leading to a period of B cell aplasia that is followed by reconstitution from precursor naïve non-class-switched B cells, which successfully pass through the checkpoints by which B cell tolerance is maintained.

### 1.3. Production of Allogeneic CAR-T

To produce allogeneic genome-edited CAR-T, circulating αβ T cells are isolated from a healthy donor (pre-screened for multiple blood-borne pathogens) using leukapheresis. T cells are activated and transduced, typically using an integrating viral vector, to achieve stable CAR expression. Additionally, genome editing technology is used to disrupt genes that mediate alloreactivity (e.g., *TRAC*) and immunogenicity (e.g., *B2M*). Edited CAR-T cells are expanded using cytokines, depleted of residual αβ TCR^+^ cells (which incur the risk of GVHD [12]) and are formulated as a cryopreserved master cell bank containing multiple dosing units, allowing for storage and transportation as required [6].

**Figure 2:**
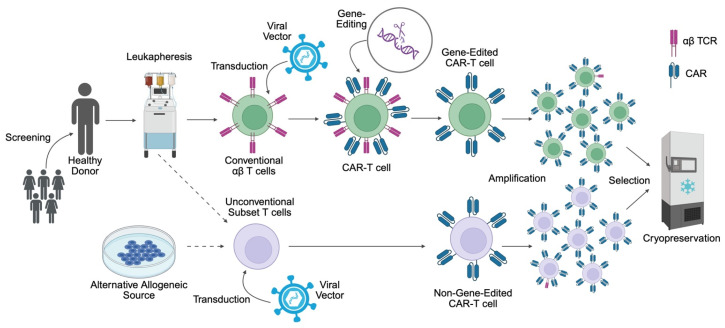
Manufacture of allogeneic CAR-T cells: The top half of the diagram depicts the manufacture of allogeneic, genome-edited αβ CAR-T from healthy donor cells, while the bottom half illustrates the manufacture of non-genome-edited CAR products, using unconventional T cell subsets (e.g., NK cells or γδ T cells) or alternative allogeneic sources (e.g., iPSCs). Created with BioRender.com.

Alternatively, specific, non-alloreactive subsets of T cells, such as NK or γδ cells, can be isolated from donor products such as peripheral blood apheresis, or expanded from other sources such as umbilical cord blood (UCB), induced pluripotent stem cells (iPSCs) or pre-existing cell lines [13]. These can then be engineered to express a CAR without the need for TCR inactivation, to produce a non-genome-edited CAR product, or can be further enhanced with gene edits, such as the prior mentioned immunogenicity-preventing disruptions, to combine the two manufacturing strategies [8]. These contrasting production processes are illustrated schematically in Figure 2.

## 2. Results

### 2.1. Genome-Edited Database Search

**Figure 3:**
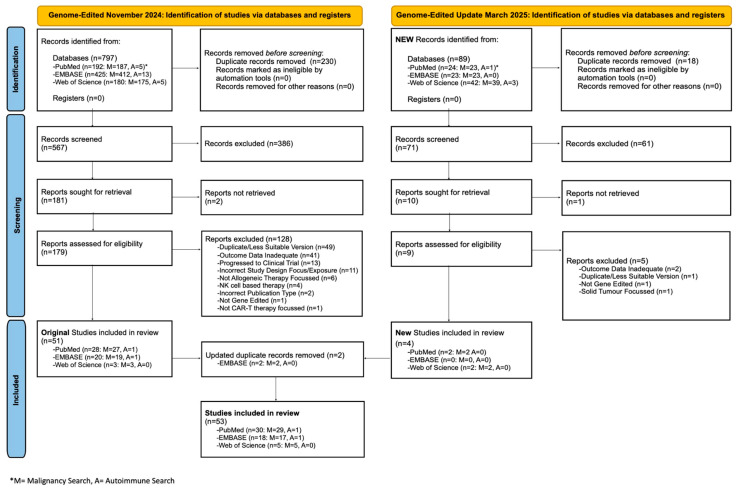
PRISMA 2020 ‘Genome-Edited’ Study Selection Flow Diagram (overleaf). The screening of articles (autoimmune- or malignancy-focused search), included and excluded studies, and databases from which articles were sourced are shown. The left side of the chart shows the initial study selection process, conducted in November 2024, while the right side shows studies that were retrieved and screened for inclusion in an updated March 2025 search. Before screening began, duplicate articles detected by Rayyan were evaluated and excluded if appropriate. Examination of titles and abstracts in the first screen led to the exclusion of 386 original and 61 new articles, for reasons such as wrong publication type (e.g., reviews), wrong intervention (e.g., autologous, NK cell or γδ CAR cells), or clearly wrong study design focus. The remaining articles were then sought, and those retrieved underwent a second, more thorough screen of full texts. This further excluded 128 original and 5 new studies. A variety of categories have been specified to summarise reasons for exclusion. Any remaining duplicates between databases, along with earlier/less data available/less focused versions of the same studies, were excluded (49 + 1 studies); a further 13 preclinical or preliminary studies were excluded as the CAR-T cell therapy that was reported had now been evaluated in clinical trials with data available. A total of 41 plus another 2 articles were excluded for ‘inadequate outcome data,’ which included lack of available results, results that were too vaguely stated, no in vivo preclinical studies (in vitro only) or claims with no supportive evidence (deemed to have inadequate risk of bias). A total of 11 articles were excluded primarily due to the study design being focused on investigating a manufacturing aspect of CAR-T, or having no target condition/group of exposures specified, as opposed to developing and trialling a CAR-T therapy to treat a specified autoimmune condition/blood malignancy; these studies did not align with the review PICO. Other self-explanatory, more specific exclusion reasons are specified in the flow diagram (e.g., wrong publication, not gene-edited, not allogeneic, not CAR-T, NK cells or solid tumour intervention). Although 51 original and 4 new articles met the inclusion criteria, 2 of the new articles were updated original studies, and hence replaced their earlier versions; hence, 53 ‘genome-edited’ studies were included overall. It should also be noted that one study could not be retrieved from the journal specified on Ovid; however, an identical publication of the abstract in another journal was retrieved, included and cited in this review [14]. Also, a peer-reviewed manuscript with the exact same title and focus was used instead of the originally retrieved abstract for one preclinical study [15].

An initial search of the use of genome-edited T cells in autoimmunity and cancer generated 797 articles (November 2024). This was narrowed down to 51 included studies after screening. The search was updated in March 2025 and identified 89 new articles, with 4 selected for inclusion, 2 of which were more up-to-date replacements of initially included studies (original versions hence removed) (Figure 3).

**Figure 4:**
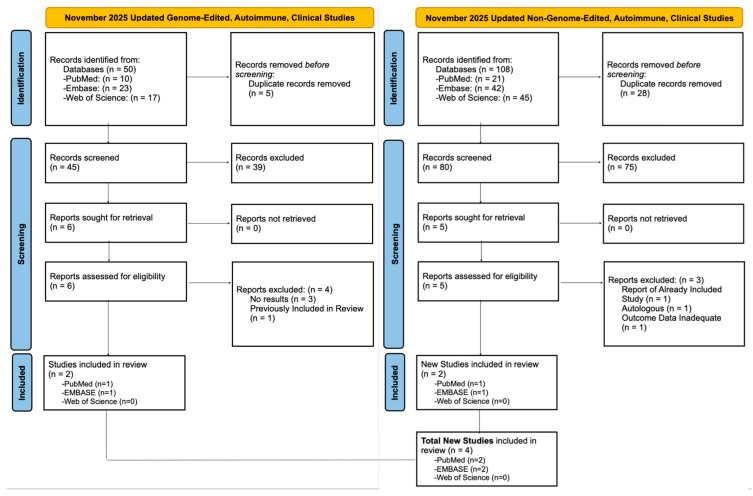
PRISMA 2020 updated search, November 2025. This search focused on clinical studies in autoimmune disease only, using either genome-edited (**left**) or non-genome-edited T cells (**right**). Selection flow diagrams indicate the number of studies identified, their database of origin and the number of included and excluded studies. Before screening began, duplicate articles detected by Rayyan were evaluated and excluded if appropriate. An initial screen of study titles and abstracts led to the exclusion of 39 genome-edited and 75 non-genome-edited studies. The remaining clinical studies were then sought for retrieval and assessed; 7 more studies were excluded owing to lack of published results, inadequate outcome data, previous inclusion in an earlier study selection, or use of an autologous CAR product. This resulted in 2 genome-edited and non-genome-edited studies being included in this review, for a total of 4 additional autoimmune clinical studies. It should be noted that for one study, a more recently published, updated conference abstract with the exact same title was used for data extraction instead of the older abstract identified on Embase [16].

A third updated search was performed in November 2025 following the initial submission of this manuscript. This search focused on clinical studies in autoimmunity alone, yielding 50 studies of genome-edited T cells, with 2 additional studies included (Figure 4, left panel).

Ultimately, this sequential screening strategy led to a final total of 55 genome-edited articles that were included in the analysis.

### 2.2. Non-Genome-Edited Database Search

**Figure 5:**
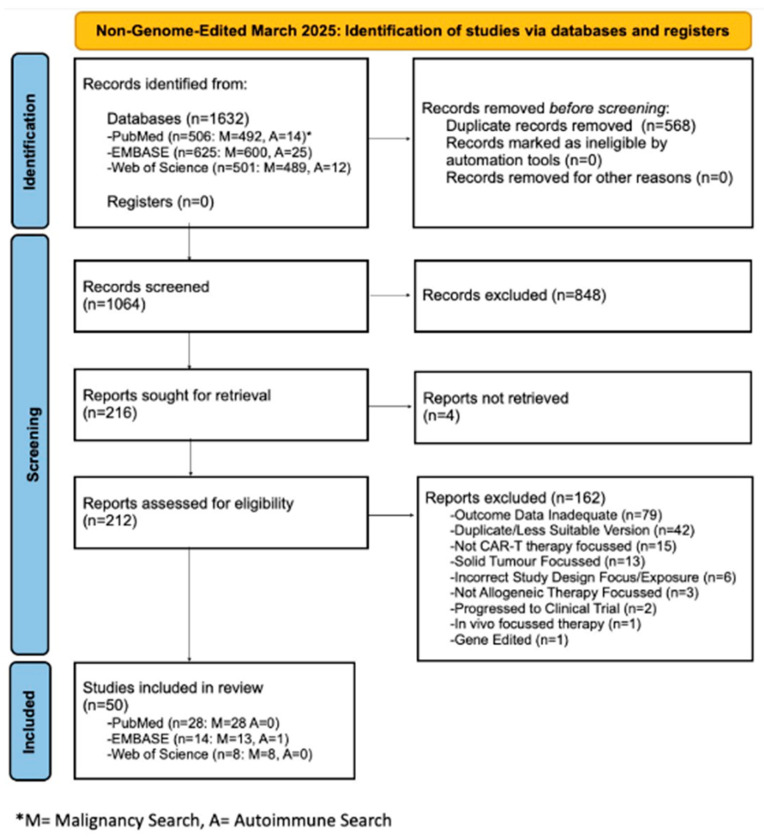
PRISMA 2020 ‘Non-Genome-Edited’ Study Selection Flow Diagram. The screening of articles (autoimmune- or malignancy-focused search), included and excluded studies, and the databases from which articles were sourced are shown. Before screening began, duplicate articles detected by Rayyan were evaluated and excluded if appropriate. A first screen, looking at titles and abstracts, excluded 848 articles, for reasons such as wrong publication type (e.g., reviews), wrong intervention (e.g., genome-edited αβ T cells), or clearly incorrect study design focus. Articles were then sought, and those retrieved underwent a second, more thorough screen of full texts. This led to the further exclusion of 162 studies. A variety of categories have been specified to summarise reasons for exclusion, as detailed in the legend to Figure 3. The only notable difference from the first review process was opposite intervention criteria, with a ‘gene-edited’ exclusion category, referring to conventional gene-edited αβ T cells, listed in this second-stage review flow diagram. Overall, 50 ‘non-genome-edited’ articles met the inclusion criteria for this review.

A search of the use of ‘non-genome-edited’ allogeneic T cells in cancer and autoimmunity was undertaken in March 2025 and generated a total of 1632 reports. These underwent a similar selection process, ultimately yielding 50 additional articles for inclusion (Figure 5).

The updated November 2025 search, which focused on clinical studies in autoimmunity, identified 108 non-genome-edited studies, with 2 additionally included in the review (Figure 4, right panel).

This sequential screening strategy led to a final total of 52 ‘non-genome-edited’ articles that were included in the analysis.

### 2.3. Data Analysis

In total, 107 studies were included in this review and all underwent data extraction. The studies were split into four relevant categories: 26 genome-edited clinical, 29 genome-edited preclinical, 10 non-genome-edited clinical and 42 non-genome-edited preclinical studies. Results collected using genome-editing and non-genome-editing approaches are presented, respectively, in Table 1 and Table 2 (clinical studies) and Supplementary Tables S1 and S2 (preclinical studies).

Firstly, for each study, a description of the CAR product, information on the CAR manufacturing methods and the study design have been recorded. When there was no available information regarding specific CAR transduction methods or gene-editing technology was unavailable, this was indicated by ‘ND (no data) method’ and ‘ND (no data) tech.’

For clinical studies, the study design information included subject conditions and quantity, and lymphodepletion regimen. The following outcomes have been reported on and presented in the results table:

- Complete Responses (CR). These were designated by the authors in autoimmunity, using relevant rheumatological measures of disease activity. For malignancy, this refers to the proportion of complete responses/complete responses with incomplete haematological recovery (CRi), with negative minimal residual disease (MRD), at day (D)28 of the study (or time period indicated).
- Incidence of Graft-vs.-Host Disease (GVHD), at any point during the study period.
- Incidence of Prolonged Cytopenia, persisting at D28 of the study (or time period indicated). Any specific reduced-cell-type data used, e.g., neutropenia, was highlighted in the tables. If the incidence of prolonged cytopenia was unavailable, with only overall cytopenia incidence or cytopenia data at an unclear time point being available, this was indicated by ‘†’.
- Incidence of cytokine release syndrome (CRS) at any point in the study period.
- Incidence of Immune-Effector Cell-Associated Neurotoxicity Syndrome (ICANS), or other CAR-T-induced neurotoxicity, reported at any point in the study period.
- Incidence of Opportunistic Infections or Infectious Reactivations, at any point in the study period.
- Fatalities, clearly stated as related to the interventional treatment administered, as well as the overall number of deaths reported across the study period in parentheses. As per the ‘Common Terminology Criteria for Adverse Events’ [17] guidance, any ‘grade 5’ adverse events (AE), indicating ‘death related to AE’, were recorded as a fatality.
- Any severe, stated as grade 3 or above (≥grade 3) [17], adverse reactions recorded were represented using bold and underlined text.
- If no data were available for an outcome, this was recorded as ND (no data).

**Figure 6:**
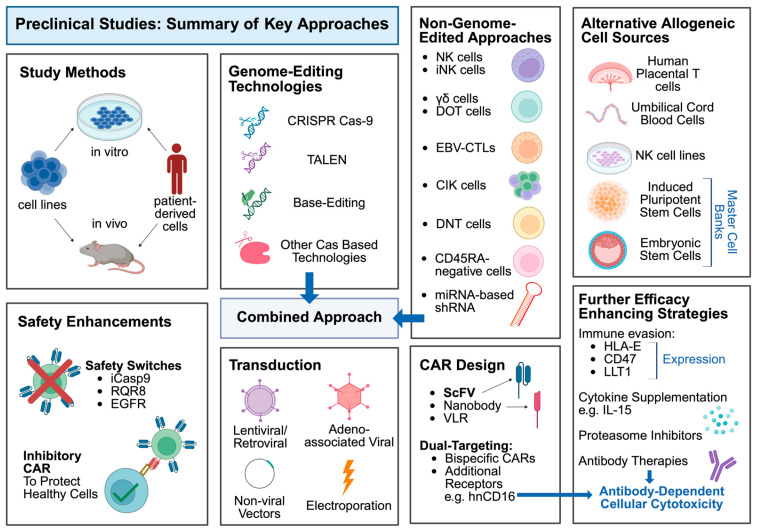
Schematic illustrating preclinical approaches used in the evaluation of allogeneic cell therapies. Created with BioRender.com.

For preclinical studies, the study design information included the main experiments and disease models used to investigate CAR products, and some of the key cell types and controls used for comparison. These approaches are summarised in Figure 6. The results were recorded as a summary of the key study findings. Outcomes reported included available in vitro and in vivo data on CAR-T expansion, antitumour efficacy and persistence, survival of xenografts, reported graft-vs.-host or alloreactivity, comparisons to conventional CAR products and any gene editing efficacy or safety outcomes (e.g., off-target effects, translocations, rearrangements). The reported key findings are particularly focused on in vivo xenografts, to present results that are likely to be most representative of what would be found in a clinical population. Furthermore, the reported key findings are focused on significant results, in comparison to relevant controls, supported by key evidence in the studies.

**Table 1:** Clinical studies using genome-edited CAR-T cells in autoimmunity (top) and cancer (bottom).

Description	Cell Design	Study Design	CR	GvHD/ Cytopenia	CRS/ ICANS	Infection	Fatalities ^1^	Ref.
**Autoimmunity**								
TyU19: An anti-CD19 CAR-T for necrotising myositis and systemic sclerosis	Healthy donor PBMC, lentiviral transduction, CRISPR Cas-9 KO of *HLA-A*, *HLA-B*, *CIITA*, *TRAC* and *PD-1*	DCSSc (n = 2), Necrotising myositis (n = 1) Flu/Cy LD	3/3	0/3 3/3 lymphopenia ^2^	0/3 0/3	ND	0/3	[18]
YTS109: An anti-CD19 targeting chimeric TCR (STAR)	Healthy donor PBMC, AAV transduction, CRISPR Cas-9 KO of *TRAC*, *PD1*, *HLA-A*, *HLA-B* and *CIITA*	SLE with nephritis (n = 5) Flu/Cy LD	2/5 ^3^	0/5 5/5 leukocytopaenia) ^4^	2/5 0/5	2/5	0/5	[19]
BRL-303: An anti-CD19 CAR-T for SLE	Healthy donor T cells, Multiplex genome-edited (ND method, no genes specified), Transduction (ND method)	r/r SLE (n = 4) Flu/Cy LD (n = 3) No LD (n = 1)	ND ^5^	0/4 1/4 (leukopaenia at 1 month)	4/4 0/4	ND	0/4	[16]
**Cancer**								
BE-CAR7: An anti-CD7 CAR-T, for relapsed T-ALL	Healthy donor PBMC, lentiviral transduction, Base editing disruption of *TRBC1 + TRBC2*, *CD52* and *CD7*	r/r T-ALL (n = 3)Flu/Cy/Alem. LD	2/3	1/3 ND	2/3 2/3	3/3	1/3	[20]
CTA101: A CD19/CD22 dual targeting CAR-T, for r/r B-ALL	CD3^+^ T cells isolated, lentiviral transduction, CRISPR Cas-9 KO of *TRAC* and *CD52*	r/r B-ALL (n = 6) Flu/Cy LD	5/6	0/6 3/6	6/6 (**1/6**) 0/6	**3/6**	(1/6 overall)	[21]
TT52CAR19: An anti-CD19 CAR-T, for paediatric r/r B-ALL	Healthy donor peripheral blood lymphocytes, lentiviral transduction (incorporating self-duplicating CRISPR sgRNA), Next generation CRISPR Cas-9 KO of *TRAC* and *CD52*	r/r B-ALL (n = 6) Flu/Cy/Alem. LD	3/6	1/6 6/6 (**6/6**) neutropenia	6/6 2/6 (**1/6**)	6/6	(4/6 overall)	[22]
UCART19: An anti-CD19 CAR-T, for adult B-ALL (CALM)	Healthy donor PBMCs, lentiviral transduction, TALEN editing of *TRAC* and *CD52*	r/r B-ALL (n = 25) Flu/Cy +/− Alem. LD	9/25	2/25 **10/25**	20/25 (**6/25**) 7/25 (**1/25**)	12/25 (**7/25**)	4/25 (14/25 overall)	[23]
U-CAR-T19 + nU-CAR-T19: Anti-CD19 CAR-Ts, for r/r B-ALL	PBMCs, lentiviral transduction, CRISPR Cas-9 (1) U-CAR-T19: *B2M* and *TRAC* KO (2) nU-CAR-T19: *HLA-A*, *HLA-B* and *TRAC* KO	(1) U-CAR-T19: DLBCL (n = 3), B-ALL (n = 3) (2) nU-CAR-T19: r/r B-ALL (n = 3) Flu/Cy/Etoposide LD	(1) 0/6 (2) 3/3 (D14 MRD neg. ^6^)	0/9 **5/9** (D14)	9/9 0/9	2/9	0/9	[24]
CD5 CAR-T: A CD5 targeting CAR-T for r/r T-ALL	PBMCs (previous transplant donor or healthy matched donors), lentiviral transduction, (EGFRt incorporated), CRISPR Cas-9 (sgRNA) KO of *CD5*	r/r T-ALL: previous transplant donor (n = 11), healthy donor (n = 5) Flu/Cy LD	16/16	11/16 16/16 (**15/16** at 1 month)	12/16 4/16	9/16 (**7/16**)	(8/16 overall)	[25]
WU-CART-007: A CD7 targeting CAR-T, for r/r T-ALL/T-LBL	Healthy Donor T cells, transduction (ND method), CRISPR Cas-9 editing to KO *CD7* and *TRAC*	T-ALL (n = 18), T-LBL (n = 8) -phase 1 (n = 13); Flu/Cy LD -phase 2 (n = 13); enhanced LD	12/23 (7/10 MRD neg. ^7^)	1/26 5/26 (neutropenia) **†**	23/26 (**5/26**) 2/26	**7/26** ^8^	1/26 (3/26 overall)	[26]
UCART19: An anti-CD19 CAR-T, for infant B-ALL	Healthy Donor T cells, lentiviral transduction (incorporated RQR8 epitope tag), TALEN-mediated KO of *TRAC* and *CD52*	Infant B-ALL (n = 2) -followed by alloHSCT Flu/Cy/Alem. LD	2/2	2/2 1/2	0/2 1/2	ND	0/2	[27]
UCD7-CAR: An anti-CD7 CAR-T, for paediatric R/R T-ALL/LBL	Healthy Donor T cell, transduction (ND method), CRISPR Cas-9 editing to KO *CD7* and *TCR*	r/r T-LBL (n = 1), r/r T-ALL (n = 4) Flu/Cy LD	4/5	0/5 ND	4/5 0/5	1/5	0/5	[28]
CTX110: An anti-CD19 CAR T, for adult r/r LBCL	Allogeneic source T cells, transduction (ND method), CRISPR Cas-9 editing to disrupt *TRAC* and *B2M*	r/r LBCL (n = 32) -2nd dose (n = 8) Flu/Cy LD	11/32	0/32 ND	18/32 3/32 (**2/32**)	**4/32**	(1/32 overall)	[29]
CB-010: An anti-CD19 CAR-T, for r/r B-NHL	Healthy Donor T cells, chRDNA CAR insertion into TRAC locus, chRDNA *TRAC* and *PD-1* KO	LBCL (n = 10), MCL (n = 3), FL (n = 2), MZL (n = 1) -3 DL Flu/Cy LD	11/16	0/16 **9/16** (neutropenia) **†**	7/16 4/16 (**2/16**)	**1/16**	ND	[30]
ET-901: An anti-CD19 CAR-T, for B-NHL	Allogeneic T cell source, transduction (ND method), CRISPR KO of undisclosed gene (*Gene X)* and *TCR*	r/r LBCL (n = 4), FL (n = 2) Flu/Cy LD	4/6	1/6 6/6 **†**	6/6 2/6	2/6	ND	[31]
UCART22 P2: An anti-CD22 CAR-T for r/r B-ALL	Donor-derived T cells, lentiviral transduction, TALEN-mediated KO of *TRAC* and *CD52*	r/r B-ALL (n = 3) Flu/Cy/Alem. LD	1/3 ^9^	ND ND	2/3 0/3	**1/3**	1/3	[32]
UCART20x22: An anti-CD20/CD22 dual-targeted CAR-T, for r/r B-NHL	Healthy donor T cells, lentiviral transduction, TALEN-mediated KO of *TRAC* and *CD52*	r/r DLBCL (n = 2), THL (n = 1) Flu/Cy/Alem. LD	2/3	0/3 ND	3/3 0/3	1/3	ND	[33]
FT819: An iPSC-derived anti-CD19 CAR-T, for r/r B cell leukaemia/lymphoma	iPSCs, transduction into *TRAC* locus (ND method), Gene-edited bi-allelic KO of TCR (ND tech.)	r/r BCL (n = 12) -regimens A: 1 dose (n = 9), B: 3 split doses (n = 3) Flu/Cy LD	ND	0/12 ND	3/12 0/12	ND	ND	[34]
RD13-01: An anti-CD7 CAR-T, for T-ALL + T-LBL	Healthy donor-derived T cells, transduction (incorporated NK32 inhibitory ligand) (ND method), CRISPR Cas-9 edited (no genes specified)	T-ALL (n = 7), T-LBL (n = 3) Flu/Cy/Etoposide LD	7/10	ND ND	10/10 (**1/10**) **1/10**	0/10	(4/10 overall)	[35]
GC027: An anti-CD7 CAR-T, for r/r T cell malignancy	Healthy donor T cells isolated, lentiviral transduction, CRISPR Cas-9 KO of *TRAC* and *CD7*	r/r T-ALL (n = 11), T-LBL (n = 1) Flu/Cy + melphalan, etoposide, or pred. LD	11/12	1/12 (post HSCT) ND	10/12 (**8/12**) 0/12	**1/12** ^10^	(2/12 overall)	[36]
UCART123v1.2: An anti-CD123 CAR-T, for AML	Healthy Donor T cells, lentiviral transduction, TALEN-mediated KO of *TRAC* and *CD52*	r/r AML (n = 17) Flu/Cy +/− Alem. LD	1/16	ND ND	17/17 (**4/17**) **1/17**	ND	(2/16 overall)	[37]
CTX130: An anti-CD70 CAR-T, for r/r TCL	Healthy Donor T cells, AAV transduction into *TRAC* locus, CRISPR Cas-9 KO of *TRAC*, *B2M* and *CD70*	PTCL (n = 22), Mycosis Fungoides or Sézary Syndrome (n = 17) Flu/Cy LD	6/39	0/39 15/39 (**14/39** neutropenia) **†**	26/39 (**3/39**) 4/39	**10/39**	0/39 (16/39 overall)	[38]
ALLO-501: An anti-CD19 CAR-T, for r/r B-NHL	Allogeneic source T cells, transduction (ND method), TALEN-mediated KO of *TRAC* and *CD52*	B-NHL (n = 46) -2nd dose (n = 7) Flu/Cy/ALLO-647 LD	18/36 (Auto CAR-T naïve)	0/46 38/46 **†**	11/46 (**1/46**) 0/46	**11/46**	1/46 (5/46 overall)	[39]
ALLO-715: An anti-BCMA CAR-T, for r/r MM	Healthy donor PBMCs, lentiviral transduction, TALEN-mediated KO of *TRAC* and *CD52*	r/r MM (n = 43) Cy/ALLO-647 +/− Flu LD	6/43	0/43 **8/43** (D56)	24/43, **1/43** 6/43	23/43, **10/43**	(10/43 overall)	[40]
ALLO-501A: An anti-CD19 CAR-T, for LBCL	Allogeneic source T cells, transduction (ND method), TALEN-mediated KO of *TRAC* and *CD52*	LBCL (n = 15) -Con: 2nd consolidation dose (n = 9) Flu/Cy/ALLO-647 LD	6/12	Con: 0/9 72% **†**	Con: 0/9 Con: 0/9	Con: **0/9**	ND	[41]
UCART19: An anti-CD19 CAR-T, for paediatric B-ALL (continued trial [31])	Healthy Donor T cells, lentiviral transduction (incorporated RQR8), TALEN-mediated KO of *TRAC* and *CD52*	r/r B-ALL (n = 5) -additional participants from original study [27] Flu/Cy/Alem. LD	3/5	1/5 2/5 (neutropenia)	5/5 (**1/5**) ND	4/5	(3/5 overall)	[42]

Bold and underlined text signifies ≥ grade 3 adverse event. ND signifies no data was available for this outcome. Abbreviations: AAV: Adeno-Associated Virus, Alem. Alemtuzumab, AML: Acute Myeloid Leukaemia, alloHSCT: Allogeneic Haematopoietic Stem Cell Transplantation, BCL: B cell Lymphoma, B-ALL: B cell Acute Lymphoblastic Leukaemia, chRDNA: CRISPR Hybrid RNA-DNA, CLL: Chronic Lymphocytic Leukaemia, Con: Consolidation dose, CR: Complete Response, CRISPR-Cas9: Clustered Regularly Interspaced Short Palindromic Repeats-CRISPR-Associated Protein 9, CRS: Cytokine Release Syndrome, CTCL: Cutaneous T cell Lymphoma, Cy: Cyclophosphamide, DCSSc: Diffuse Cutaneous Systemic Sclerosis, DL: Dose Level, DLBCL: Diffuse Large B cell Lymphoma, EGFRt: Truncated Epidermal Growth Factor, FL: Follicular Lymphoma, Flu: Fludarabine, GVHD: Graft-vs.-Host Disease, ICANS: Immune-Effector Cell-Associated Neurotoxicity Syndrome, iPSC: Induced Pluripotent Stem Cells, KO: Knockout, LBCL: Large B cell Lymphoma, LD: Lymphodepletion, LN: Lupus Nephritis, MCL: Mantle Cell Lymphoma, MM: Multiple Myeloma, MRD neg.: Minimal Residual Disease-Negative, MZL: Marginal Zone Lymphoma, NK: Natural Killer Cell, ND: No data, ND method: No specified transduction method, ND tech.: No specified gene-editing technology, NHL: Non-Hodgkin’s Lymphoma, PBMC: Peripheral Blood Mononuclear Cells, Pred.: Prednisolone, PTCL: Peripheral T cell Lymphoma, Ref.: Reference, r/r: Relapsed or Refractory, SLE: Systemic Lupus Erythematosus, STAR: Synthetic T cell and Antigen Receptor, TALEN: Transcription Activator-Like Effector Nucleases, T-ALL: T cell Acute Lymphoblastic Leukaemia, T-LBL: T cell Lymphoblastic Lymphoma, TCL: T cell Lymphoma, TCR: T cell Receptor, THL: Triple-Hit Lymphoma, UCART: Universal CAR-T cells. ^1^ Fatalities attributable to CAR-T are shown above, and overall fatalities are shown below. All fatalities were inherently a severe adverse outcome, but have not been recorded in bold or underlined since not all were necessarily stated as attributable to a ≥grade 3 adverse event. ^2^ Despite being present at the Month 1 follow-up, this lymphopenia was considered ‘temporal’ in this autoimmune CAR-T study. ^3^ While 2/5 were in complete remission at 3 months, 5/5 met the primary efficacy endpoint of SLE responder index 4 (SRI-4) criteria. ^4^ Although it was referred to as transient, all 5/5 subjects experienced leukocytopenia ‘> D28’ hence recorded as prolonged cytopenia by this review’s definition. ^5^ Data on D28 complete responses was not provided, although 4/4 subjects met the Systemic Lupus International Collaborating Clinics (SLICC) criteria for sustained response (SRI-4), and 1/4 subjects were in drug-free remission at 3 months (remaining 3/4 on low dose glucocorticoid maintenance). ^6^ For this study, only D14 MRD-negativity data was available (rather than D28 CR) and is recorded as such. Effectiveness of nU-CAR but not U-CAR attributed to lack of NK cell recognition. ^7^ Only 10/12 CR subjects had MRD data available, 7/10 of whom were MRD undetectable. ^8^ The figure of **7/26** patients suffering from infections (grade 3 or above) was obtained by cumulating the ‘5 patients’ suffering from ‘grade 3 or 4 infection events’ with ‘2 grade 5 events secondary to invasive fungal infections’. However, this data was slightly ambiguous with regard to patient overlap. ^9^ Instance of MRD negative ‘MFLS’ (Myeloid-free leukaemic state) was not categorised as a complete response (since other cases of CR/CRi had been specified separately). ^10^ Recorded in bold and underlined, as clearly stated in the study text, that this was a ‘severe’ infection that led to a fatality. **†** Signifies that the incidence of prolonged cytopenia, or the time period for any recorded cytopenia, was unavailable; therefore, the incidence of ‘overall cytopenia’ has been recorded (not necessarily prolonged).

**Table 2:** Clinical studies using Non-genome-edited CAR cells in autoimmunity (top) and cancer (bottom).

Description	Cell Design	Study Design	CR	GvHD/ Cytopenia	CRS/ ICANS	Infection	Fatalities	Ref.
**Autoimmunity**								
QN-139b: CD19 BCMA dual CAR targeted iPSC-derived NK cells	iPSC engineered using CRISPR Cas-9 to express CD19 and BCMA CARs in addition to KO by cytosine base editing of *B2M*, *CIITA*, *CD16* and over-expression of HLA-E, HLA-G, IL-2 receptor fusion and EGFRt. Differentiated thereafter into NK cells	DCSSc (n = 1) Flu/Cy LD	PR ^1^	0/1 1/1 ^2^	0/1 0/1	1/1	0/1	[43]
Allogeneic CD19 CAR-NK: An anti-CD19 CAR-NK for SLE	Allogeneic Source, CAR-NK (ND methods)	r/r SLE (n = 24) Flu/Cy LD	8/12 (DORIS at 12 months) ^3^	0/24 ND	2/24 ^4^ 0/24	ND	ND	[44]
**Cancer**								
CYAD-211: An anti-BCMA CAR-T (engineered using miRNA-based shRNA), for r/r MM	Healthy donor PBMCs, retroviral transduction (encoding miRNA-based shRNA against CD3ζ)	r/r MM (n = 12) Flu/Cy LD	0/12 (3/12 PR)	0/12 2/12 (neutropenia) **†**	1/12 0/12 (CRES)	5/12 (**2/12**)	(1/12 overall)	[45]
FT596: An iPSC-derived anti-CD19 CAR-NK for BCL	iPSCs, lentiviral transduction (incorporated hnCD16 + IL-15-RF and NK-optimised CD19 CAR with NKG2D transmembrane domain, 2B4 co-stimulatory domain and CD3ζ activation domain), differentiated into NK cells	BCL (n = 86) regimen A (without rituximab, n = 18) or B (with rituximab, n = 68) Flu/Cy LD	25/68 (efficacy reported for B alone)	0/86 **17/81**	10/86 0/86	**17/86**	0/86 (43/86 overall)	[46]
CD33 CAR-NK: An anti-CD33 CAR-NK for r/r AML	NK cells from healthy donor UCB, lentiviral transduction	r/r AML (n = 10) Flu/Cy LD	6/10	0/10 10/10 (**5/10**) (neutropenia) **†**	1/10 0/10	3/10 (**1/10**)	(9/10 overall)	[47]
CNTY-101: An anti-CD19 iPSC-derived CAR-NK for B-NHL	iPSC, CRISPR MAD-7 mediated KO of *B2M* and *CIITA*, CRISPR mediated *HLA-E* expression, transduction (incorporated secreted IL-15 and EGFR safety switch) (ND method), differentiated into CAR-NK [48] ^5^	DLBCL (n = 5), FL (n = 1), MZL (n = 1) Chemo. LD	2/7	0/7 ND	2/7 0/7	ND	ND	[49]
NKX101: An anti-NKG2D ligand CAR-NK for r/r AML	Healthy donor NK cells, transduction (incorporated membrane-bound IL-15) (ND method)	r/r AML (n = 6) Flu/Ara-C LD	3/6	0/6 **3/6** (neutropenia) **†**	0/6 0/6	**3/6**	ND	[50]
ADI-001: An anti-CD20 CAR-γδ for BCL	Allogeneic γδ T cells, transduction (ND method)	LBCL (n = 8), MCL (n = 1) Flu/Cy LD	7/9	0/9 ND	2/9 1/9	**1/9**	0/9 (1/9 overall)	[51]
CD19-CAR NKT (ANCHOR): An anti-CD19 CAR- iNKT for B-NHL + B-ALL	NK T cells from leukapheresis product, transduction (incorporated IL-15, and encoding shRNA targeting *B2M* and *CD74*) (ND method)	B-NHL (n = 4), B-ALL (n = 1) Flu/Cy LD	2/5	ND ND	1/5 ND	ND	ND	[52]
19-28z CAR EBV-CTLs: An anti-CD19 CAR-EBV-CTL for r/r B cell malignancy	EBV-CTLs, from primary HSCT donors (n = 4) or 3rd Party donors (n = 6), transduction (ND method)	B-ALL (n = 5), Burkitt’s (n = 1), CLL (n = 1), PMBCL (n = 2), DLBCL (n = 1) ND on LD	7/10	1/10 ND	0/10 0/10	ND	ND	[53]

Bold and underlined text signifies ≥ grade 3 adverse event. ND signifies no data was available for this outcome. Abbreviations: +/−: with or without, AML: Acute Myeloid Leukaemia, Ara-C: Cytarabine, B-ALL: B cell Acute Lymphoblastic Leukaemia, BCL: B cell Lymphoma, B-NHL: B cell Non-Hodgkin Lymphoma, Chemo: Chemotherapy (no drug specified), CLL: Chronic Lymphocytic Leukaemia, CR: Complete Response, CRES: CAR-T cell-Related Encephalopathy Syndrome, CRISPR-Cas9: Clustered Regularly Interspaced Short Palindromic Repeats-CRISPR-Associated protein 9, CRS: Cytokine Release Syndrome, Cy: Cyclophosphamide, DLBCL: Diffuse Large B cell Lymphoma, DL: Dose Level, EBV-CTL: Epstein–Barr Virus Specific Cytotoxic T-Lymphocytes, EGFR: Epidermal Growth Factor Receptor, EGFRt: Truncated Epidermal Growth Factor, FL: Follicular Lymphoma, Flu: Fludarabine, γδ: Gamma-delta, GVHD: Graft-vs.-Host Disease, HSCT: Haematopoietic Stem Cell Transplantation, hnCD16: High-Affinity, Non-Cleavable CD16, ICANS: Immune-Effector Cell-Associated Neurotoxicity Syndrome, IL-15-RF: IL-15/IL-15-Receptor Fusion Molecule, iNKT: invariant NKT, iPSC: Induced Pluripotent Stem Cell, KO: Knockout LBCL; Large B cell Lymphoma, LD: Lymphodepletion, MCL: Mantle Cell Lymphoma, miRNA: micro-RNA, MM: Multiple Myeloma, MZL: Marginal Zone Lymphoma, Nb: Nanobody, NK: Natural Killer, ND: No Data, ND method: No specified transduction method, PBMC: Peripheral Blood Mononuclear Cells, PMBCL: Primary Mediastinal B cell Lymphoma, PR: Partial Response, Ref.: Reference, r/r: relapsed/refractory, shRNA: short hairpin RNA, TCR: T cell Receptor, UCB: Umbilical Cord Blood. ^1^ Data on complete responses not provided, but clinical improvement scores indicated improvement consistent with a partial response. ^2^ B cell Cytopenia present at Month 1 indicated by graphs of immune cell recovery. ^3^ Met Definition of Remission of SLE (DORIS) upon follow-up at least 12 months. ^4^ The conference abstract, although stating 2 of 18 patients suffered from grade 1 CRS, also stated 8% CRS incidence (equivalent to 2/24), hence recorded as 2/24 (24 patients treated). ^5^ Limited information was provided in this article on the CAR-T product design and manufacture; therefore, information from another preclinical study of CNTY-101 product for autoimmune disease, was provided. † Signifies that the incidence of prolonged cytopenia, or the time period for any recorded cytopenia, was unavailable; therefore, the incidence of ‘overall cytopenia’ has been recorded (not necessarily prolonged).

## 3. Discussion

For allogeneic CAR-T cells to be successfully commercialised as a therapy for autoimmune diseases, there is a need for a highly safe and effective product that can be manufactured using a large-scale, cost-effective process. Here, we set out to evaluate strategies implemented so far to achieve these aims, evaluating both gene-edited and non-genome-edited approaches. Given the paucity of clinical data in the setting of autoimmune disease, data were also collected on cancer in an effort to learn from principles established in that field.

### 3.1. A Summary of the Findings

**Figure 7:**
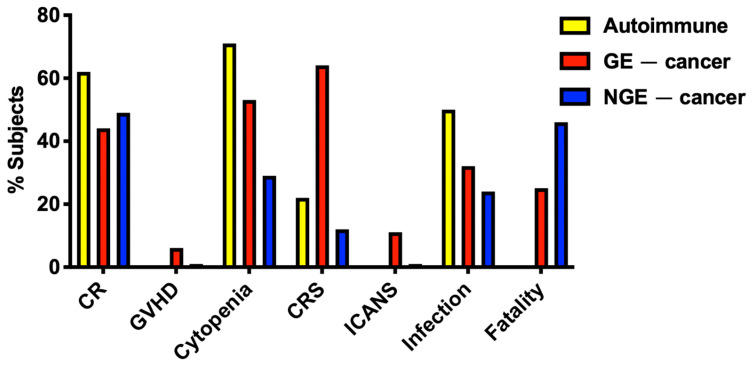
Efficacy and safety of allogeneic CAR-based treatment. Data are shown for 21 patients with autoimmune disease (as response data were not presented for all patients with autoimmune disease) and 455 patients with cancer. The latter are subdivided into those treated with genome-edited (GE, n = 328) or non-genome-edited (NGE, n = 127) cells. Percentage of treated subjects who achieved complete remission (CR) of their disease, experienced toxicity (GVHD, cytopenia, CRS, ICANS, infection) and all-cause fatality are also shown.

Clinical studies are shown in Table 1 (genome-edited cells) and Table 2 (non-genome-edited cells). The efficacy and safety of these approaches in autoimmune disease and cancer are summarised in Figure 7. Such data have only been reported for 21 patients with autoimmune disease, with 13 complete remissions (CRs) of disease and marked improvement in disease status reported for additional subjects, albeit with limited follow-up. These therapies have been well tolerated in patients with autoimmune disease, with lymphodepletion-related cytopenias being the dominant toxicity seen. Graft versus host disease has not been evident, while cytokine release syndrome has been infrequent and generally of lower grade than has been seen in cancer. These safety issues are discussed further below.

### 3.2. Genome-Editing to Address Immunological Barriers Imposed by Allogeneic T Cells

The HLA-restricted TCRs of donor-derived allogeneic cells have the potential to engage healthy host cells, causing GVHD. To mitigate this risk, genome-editing strategies can be deployed to knockout a single component of TCR/CD3, thereby preventing cell surface expression of the entire complex [12]. This strategy has been used across all allogeneic CAR-T trials using genome-edited cells. Most targeted the TCR alpha constant locus (*TRAC*) [18], with some instead targeting TCR beta constant genes (*TRBC1/2*), using a guide RNA to target sites of homology within both loci [20,54]. Using this approach, GVHD was infrequently observed in both clinical trials of cancer and autoimmune disease and was mostly mild in severity (grade one or two) with cutaneous [23] or occasional intestinal [25] involvement, resolving fully without intervention or with corticosteroid therapy [26,27].

On the other hand, the recognition of foreign ‘mismatched HLA’ molecules on infused allogeneic CAR-T cells can render them susceptible to host-mediated rejection. To reduce this risk, knockout of the *B2M* gene has been widely used to eliminate cell surface expression of all HLA-I molecules. However, certain HLA-I antigens, notably HLA-C and HLA-E, can inhibit NK cell activation via engagement of inhibitory KIR (killer cell immunoglobulin-like receptors) and CD94-NKG2A, respectively, meaning that global knockout of *B2M* can paradoxically facilitate NK cell-mediated rejection of these cells. In contrast, selective editing of HLA-A and HLA-B preserves NK cell inhibition, maintaining therapeutic efficacy against B-ALL without rejection of allogeneic CAR-T [24]. This strategy was deployed in both the TyU19 and YTS109 products for autoimmune disease, in which healthy donor PBMC were edited to knock out *HLA-A*, *HLA-B* and *CIITA*, which are required for HLA-II expression [18,19]. Other strategies to minimise the impact of HLA mismatch while maintaining NK immune tolerance have been demonstrated in preclinical trials of ‘hypoimmune’ CAR-T. Examples include the expression of HLA-E [55], B2M-HLA-E+ polyprotein [56] or LLT1 [57] to maintain NK cell tolerance and the use of CD47 overexpression [58,59,60] or ‘anti-CD47-nanobody Fc fusion’ [61] secretion to prevent phagocytosis. Alternatively, a proprietary gene labelled ‘gene X’ (an activation-induced cell death mediator) has been identified, the disruption of which also demonstrated clinical efficacy as a strategy for achieving prolonged immune persistence [31].

Genome editing can also be implemented to further enhance the effectiveness and persistence of CAR-T cells. Conceptually, this would be useful in autoimmune disease to ensure a deep B cell depletion that persists long enough for ‘immune reset’ to occur. Disruption of the T cell-inhibitory PD-1 receptor [62] is a strategy that was implemented to reduce CAR-T cell exhaustion in subjects with relapsed, refractory B cell lymphoma [30]. It has also been used in the treatment of autoimmune disease, where deep B cell depletion was sustained for two months to induce remission in all patients [18]. Furthermore, genome editing was successfully implemented to prevent the fratricide of CAR-T products used for the treatment of T cell malignancy, by knocking out the CAR target antigen (e.g., *CD7* [20] and *CD70* [38]). Further enhancement of CAR-T persistence could be achieved by the knockout of epigenetic modifiers, such as *DNMT3A* [63].

### 3.3. Genome-Editing Technologies

‘CRISPR Cas-9’ is a widely used genome editing technology employed across a range of studies and relies on single-guide RNA molecules to target specific DNA, where a double-stranded break is made. This break is then repaired by ‘non-homologous end joining,’ an imprecise cellular mechanism whereby ‘indels’ lead to gene mutation and inactivation. Alternatively, in the presence of an exogenous DNA template, homology-directed repair can occur, with precise insertion of the replacement DNA. A key risk, however, is the occurrence of ‘off-target effects’, whereby genome edits occur at undesirable locations, potentially causing dangerous genomic rearrangements or oncogene activation [64].

‘TALEN’ editing is a related technology that similarly induces a double-stranded DNA break. It has a higher degree of specificity and is less prone to off-target effects than CRISPR. However, it is less efficient, harder to design and more labour intensive [65], potentially hindering commercial competitiveness. Other developments to enhance CRISPR genome editing’s specificity and safety include ‘Cas-CLOVER’ [66] or ‘Cas12a DNA-RNA hybrid CRISPR’ [56]. Despite the enhanced accuracy, the induction of a double-stranded break still carries the risk of unintended genomic alterations and chromosomal recombination [67].

Base editing instead uses guided cytidine and/or adenine deamination to alter individual nucleotides without introducing a double-stranded DNA break. This represents an attractive solution, with positive clinical trial results showing no likely off-target effects [20]. Furthermore, preclinical testing has shown no increase in translocations from background unedited levels (in contrast to CRISPR Cas-9), with the ability to efficiently introduce four simultaneous gene edits in a ‘GMP (good manufacturing process)-compliant process’ [68]. This renders base editing a highly promising and safe technology to generate allogenic CAR-T cell therapies, with potential for even more cost-effective, ‘large-scale manufacturing’, using cheaper, efficient ‘circRNA’ guides [69].

### 3.4. Non-Genome-Edited Strategies Using Unconventional Cell Types

The principle behind ‘non-genome-edited’ CAR therapy is the prevention of alloreactivity without the need for ab TCR editing. Transduction of specific lymphocyte sub-populations, which, unlike αβ cells, lack a reactive TCR, in principle mitigates the risk of GVHD without genetic disruption [70]. These ‘unconventional’ cells maintain their inherent cytotoxicity, instead relying on MHC-independent targeting strategies, rendering them attractive candidates for the development of allogeneic therapies [8]. Across the range of preclinical studies, CAR engineering has generally resulted in a further enhancement of efficacy against target cells in vitro and in vivo, with some now being advanced into clinical development. Together, this highlights the promising potential of non-genome-edited CAR therapies across a range of different cell subsets, such as γδ T cells, iNKT cells and NK cells [70].

The majority of non-genome-edited studies summarised in this review employed Natural Killer (NK) cells. These cells are integral to the innate immune system and can deploy a range of lytic mechanisms, including release of cytokines, cytotoxic granules, death ligand upregulation and antibody-dependent cell-mediated cytotoxicity. Hence, further enhancing cell targeting through CAR transduction on the background of such inherent cytotoxicity has the potential to create a particularly effective cell-based therapy [71]. CAR-NK cells have hence been successfully implemented in five non-genome-edited clinical trials, with no cases of GVHD reported [46,47,49,50]. However, despite some promising early clinical responses demonstrated, it should be noted that in an update statement regarding the NKX101 product, which originally had achieved 3/6 MRD negative complete responses (with 4/6 CR/CRi overall) [50], there was only one complete response reported in the next cohort of 14 patients, which has lead to the product being deprioritized by Nkarta [72].

In an alternative approach, one clinical trial [56], along with numerous preclinical studies, has highlighted the use of CAR-engineered invariant Natural Killer (iNK)T cells. These cells are named for their invariant TCRα chain that does not promote GVHD, but instead facilitates TCR-mediated targeting of CD1-expressing cells, as well as enabling clinically useful expansion through CD1-expressing antigen-presenting cells incubated with α-galactosyl ceramide glycolipid [73]. When engineered with an appropriate CAR, this imparts the cells with a dual-targeting specificity via the CAR and TCR.

Another cell type being keenly investigated for CAR therapies is gamma-delta (γδ) T cells, which also have a natural role in immune surveillance. These cells possess a γδTCR (broadly divided into δ2 and non- δ2 (mainly δ1) subtypes) that recognises antigens often associated with cellular injury or tumour transformation. Since the γδTCR is not HLA-restricted, this again limits the potential for the induction of GVHD [74]. γδ cells also express Natural Killer receptors (NKRs), which are non-TCRs that similarly recognise ‘stress-induced surface molecules’, and show an association with protection against infection [74]. Enhanced γδ T cell reconstitution after HSCT has been correlated with lowered rates of infection [75]. The results from a clinical study of ‘ADI-001’, an anti-CD20 CAR-γδ in 8 BCL patients, showed no cases of GVHD, and minimal notable adverse effects (only one grade 3 AE, a case of adenoviraemia), with similar success seen in preclinical studies of CAR-γδ products [51]. Furthermore, another preclinical study investigated ‘CD123CAR-DOTs’, made from ‘Delta-one T cells’, Vδ1^+^γδ enriched cell product, with the effective tumour control being significant compared to controls [76]. Delta-one T cells have the additional advantages of low exhaustion marker expression (e.g., PD-1) [77], increased expression of tissue residency markers [78], ability to undertake immune surveillance via CD1d [79] and upregulated NK-like cytotoxic receptors compared to other γδ cells [76]. Additional γδ T cell subsets (e.g., δ3 cells) can recognise the MR1 antigen-presenting molecule [80], providing an additional mechanism to detect metabolically stressed cells.

Epstein–Barr Virus-specific cytotoxic T-lymphocytes (EBV-CTL) are a category of T cell that specifically target EBV-infected cells, making them of use in the treatment of EBV-related malignancies such as Hodgkin’s, Burkitt’s and diffuse Large B cell lymphomas [81]. There are also several autoimmune diseases linked with EBV, with rheumatoid arthritis and multiple sclerosis, for example, correlating specifically with chronic B cell infection [82], potentially making EBV-CTLs naturally suited to autoimmune therapy [81]. Previous clinical studies of donor-derived prophylactic EBV-CTL infusion following HSCT have shown that the frequency of GVHD was limited [83], suggesting their potential as non-genome-edited allogeneic CAR T cell hosts. In keeping with this, 19-28z CAR EBV-CTLs derived from a mix of previous HSCT and third-party donors were trialled in a clinical study for B cell malignancies in patients post-HSCT. They were successful in inducing 7/10 CRs, with 5/6 of these being third-party cell products, while only one case of biopsy-positive GVHD was described [53]. Although this resolved with a course of topical steroids [53], it does highlight the potential alloreactivity of these cells, along with their ‘risk of allorejection’ [83]. Moreover, a preclinical study of GD2-CARrejTs for Extranodal NK/T cell lymphoma highlighted the need for further gene editing of the effector cells to produce a safe allogeneic clinical product [84].

There were a variety of other rarer cell subsets that were investigated preclinically as allogeneic CAR therapy host cells. Cytokine-induced killer (CIK) cells are a combined mix of T, NK and NK-T cells, which together exert ‘non-MHC-restricted cytotoxicity’. CIK/63.28.z, transduced with an anti-CD19 CAR, despite significantly prolonging symptom-free survival in xenograft models, did unfortunately lead to 2/7 mice being sacrificed with signs of GVHD [85]. Another noteworthy T cell subpopulation is Double-Negative T (DNT) cells that express CD3 but lack CD4 or CD8 co-receptor expression, limiting alloreactivity and GVHD potential. Directed with a CD4-specific CAR for T cell malignancies, they significantly improved xenograft tumour control compared to non-transduced control cells, which was then further enhanced by idelalisib pretreatment. Although such pretreatment similarly improved the effector function of conventional CD4-specific CAR-T cells, these mice showed signs of acute GVHD with significantly higher sickness scores compared to the CD4-CAR-DNT equivalents [86]. A final effector cell investigated was CD45RA-negative T cells. CD45RA is expressed on naive cells and is associated with alloreactivity. By contrast, expression of the alternative CD45RO isoform found on favourable memory cells has been linked to a lower risk of GVHD [70], while maintaining T cell effector function [87]. In a preclinical study of anti-CD19 CAR-CD45RA negative cells, they significantly improved in vivo tumour suppression and survival compared to NT controls, while CAR-CD45RA^+^ cells achieved similar antileukaemic activity, but led to severe xenogeneic GVHD and death of all mice before D50 [87].

While many of the aforementioned approaches help to reduce the risk of GVHD, there is still the issue of host-vs.-graft rejection to consider. This is especially the case once the host immune system recovers after initial lymphodepletion, leading to a lack of in vivo persistence and potentially limiting the safety of these therapies [70]. Some non-genome-edited CAR products, such as CAR33-NKT, have shown in vitro resistance to T cell- and NK cell-mediated rejection with low-level related HLA/NK surface molecule expression [88]. Similarly, allogenic DNT cells have also demonstrated their unique preclinical resistance to rejection [89]. Nonetheless, for clinical application in often more immunocompetent autoimmune patients, therapies potentially require further modification to prevent allorejection, as well as enhance efficacy [70].

To achieve enhanced efficacy, a number of preclinical and clinical studies sought to combine a non-TCR edited ‘unconventional’ (e.g., non-HLA-restricted) base cell with additional gene-edited enhancements to create an enhanced CAR therapy. Commonly used edits to mitigate the risk of T cell-mediated rejection included knockout (KO) of *CIITA* [48,49,90,91] and/or *B2M* [48,49,90,91,92]. Additionally, a variety of strategies were implemented to avoid the NK-mediated rejection linked to removing *B2M* expression, such as CRISPR-mediated HLA-E site-specific insertion and expression [48], transduction of a piggyBac vector encoding HLA-E [92], or ablation of CD54 and CD58 adhesion ligands, preventing host NK cells from binding [90]. In some cases, multiple gene edits were combined in an effort to achieve this, illustrated by upCAR-NK cells in which seven modifications were incorporated in iPSCs. These consisted of the combined expression of a CAR and CD24 (a macrophage ’don’t eat me’ signal), as well as knockout of *PDCD1* and *CTLA4* immune checkpoint inhibitors. Additionally, the *TRA* (TCR alpha) locus was edited to eliminate the possibility of unwanted T cell differentiation and thereby mitigate GVHD risk [91]. Other gene edits implemented were for fratricide prevention (e.g., CD5 KO) [93] and CD38 KO to allow combination therapy with the anti-CD38 monoclonal antibody daratumumab [94]. Although evidently useful to potentiate efficacy and rejection resistance, such complex gene editing strategies carry the previously discussed risks, as well as added manufacturing costs, which require consideration for commercial feasibility.

On the other hand, a number of recently described alternatives to gene editing enhancement of T cells have emerged. Illustrating this is the fully non-genome-edited, allogeneic CAR product, CYAD-211 [45]. This T cell product expresses an anti-BCMA CAR together with a vector-embedded shRNA that targets CD3ζ, a component of the TCR/ CD3 complex. Although successful in preventing GVHD, with 0/12 cases, efficacy in patients with multiple myeloma was somewhat limited. Despite CAR-T expansion across all three dose levels, only 3/12 partial responses were observed, with rapid onset of CAR T cell exhaustion (engraftment 3–4 weeks). This limitation was attributed to probable host vs. graft rejection. In another clinical study, this technology was implemented to prevent rejection of iNKT cells, using shRNA to target *B2M* and *CD74* for HLA I and II downregulation [52]. This suggests that non-genome-edited technologies, such as RNA interference-based approaches, could be used to create a fully non-genome-edited, rejection-resistant CAR product [45].

### 3.5. Safety Considerations

Cytokine release syndrome (CRS) and Immune-Effector Cell-Associated neurotoxicity syndrome (ICANS) occurred in many studies, with both often reported. Promisingly, most cases were low grade, although 11/25 and 6/25 gene-edited clinical trials reported at least one case of ≥grade 3 (more severe) CRS or ICANS, respectively. Although the number of patients with autoimmune disease is low, these toxicities appear to be less frequent and severe in this setting than has been the case in cancer. Many cases were effectively managed with treatments including tocilizumab, glucocorticoids and oxygen supplementation [21], or in one reported case, an additional anti-cytokine agent (anakinra) [38]. However, some subjects needed intensive care admission [22,23], and one trial reported two grade-5 (death related to adverse event [17]) CRS occurrences [37]. Notably, across the eight non-genome-edited clinical studies, there were no reported grade-3 or greater events of either CRS or ICANS, with a trend towards low overall CRS incidence and only one reported case of grade-1 ICANS across all eight studies. Although classified as only grade 1, there was a case of a subject hospitalised with CRS in the CYAD-211 study, which was consequently considered a serious adverse event [45]. The suggested explanations for the safety of these products include the lack of vigorous clonal expansion with NK cell products, and lower-level inflammatory cytokine release, especially with γδ cells compared to αβ counterparts [95]. In keeping with this, one included study reported undetectable ‘IL6, IFNγ, and TNFα’ release with a γδ CAR-T product [96].

A high prevalence of prolonged cytopenia and related opportunistic infections (often ≥grade 3) occurred across gene-edited and non-genome-edited clinical studies. Although expected with the use of lymphodepleting chemotherapy, cytopenias were very common, sometimes stated as the most frequent adverse event [40,52]. Furthermore, there were several infection-related deaths reported in gene-edited studies, such as from fungal infection [20,26], HHV-6 [29], Klebisella pneumoniae [25] encephalitis, Epstein–Barr Virus [25], adenoviral hepatitis [40], pneumonia [36,38,39,40] and sepsis [32,38,40], as well as a grade 5 HSV infection in a non-genome-edited clinical study [45]. Despite only ‘temporal lymphopenia’ and minimal adverse effects in the TyU19 autoimmune study [18], the occurrence of more severe side effects in the cancer studies mandates enhanced safety measures in future autoimmune trials.

CAR-T ‘suicide genes’ [97] enable the pharmacological elimination of the transduced cells, providing a reassuring fallback safety measure. One commonly used approach entails the co-expression of RQR8 in CAR-T cells. This marker incorporates rituximab epitopes to facilitate rituximab-mediated depletion [27] and has been validated in one clinical study [42]. However, there were reported issues with lowered RQR8 expression levels compared to co-expressed CARs, raising questions about the sensitivity of this approach to enable rituximab-mediated depletion [27]. Moreover, a related safety switch mechanism employing truncated epidermal growth factor receptor (EGFRt) failed to induce CAR-T depletion in four patients with uncontrollable infections, upon administration of anti-EGFR antibody [25]. Another (short) epidermal growth factor (sEGFR) was co-expressed in the preclinical CAR-NK product, CNTY-101 and enabled effective depletion with cetuximab, although this was only trialled in vitro [48]. Alternatively, ‘Inducible Caspase9 (iCasp9)’ is a highly effective cell depletion approach, activated by dimerisation in the presence of the chemical dimeriser AP1903 (rimiducid) or AP20187 [66,98,99], with as little as 10 nM of rimiducid resulting in effective CAR T cell apoptosis [98]. When used clinically, this method has achieved rapid depletion of CAR T cells, abrogating GVHD [100] and toxicity such as severe, steroid-refractory ICANS [101]. The deployment of iCasp9 could therefore be a promising safety strategy for use in allogeneic CAR-T cells. Furthermore, the recent development of a rapamycin-triggered iCasp9 system allows the use of this technology in conjunction with a readily available, licenced pharmaceutical [102].

With regard to ‘non-genome-edited’ unconventional cell source therapies, a key advantage of certain subsets, such as NK cells and γδ T cells, is their expression of multiple receptors and adaptations that allow for the additional targeting of unhealthy target cells. Illustrating this, CD19 CAR- γδ T cells were able to target CD19-negative tumour cells in vitro, an advantageous adaptation for overcoming ‘antigen loss’ and evasion [103]. Although clearly advantageous for cancer therapy, and potentially able to enhance autoreactive cell deep depletion, this non-specific targeting capability does raise hypothetical concerns regarding off-target cell lysis by autoimmune candidate therapies. Reassuringly, in vitro studies have shown that certain CAR-NK therapies preferentially lyse CAR-target cells compared to healthy ones [104], as well as mediating comparable, minimal lysis of healthy cells compared to non-transduced NK controls [105]. A particularly noteworthy safety strategy that sought to eliminate off-target concerns entails the use of a tricistronic ’OR-NOT’ logic-gated CAR, which included a protective inhibitory CAR, co-expressed alongside a typical ’activating’ anti-FLT3/anti-CD33 CAR. In this case, the inhibitory CAR was targeted against endomucin antigens, found uniquely on healthy HSCs, thereby protecting them from lysis. When the ’OR-NOT’ CAR was expressed in NK cells (with IL-15 support), they achieved both a significant reduction in tumour burden and the preservation of healthy cells in vivo [106]. This could represent a very promising safety strategy for autoimmune therapies if a biomarker only present on healthy cells and not autoreactive cells could be delineated as an inhibitory CAR target.

### 3.6. Lymphodepletion

Lymphodepletion to deplete host immune cells is essential for allogeneic CAR-T expansion [107]. Review data collected highlights a trend to indicate that the intensity of lymphodepletion was linked to improved patient outcome. Illustrating this, one trial showed that more intensive fludarabine and cyclophosphamide conditioning led to ‘seven times higher’ CAR-T expansion [26]. In another case, while fludarabine and cyclophosphamide alone were insufficient [37], additional incorporation of alemtuzumab, an anti-CD52 monoclonal antibody [108], was required to achieve CAR-T clinical responses [23]. In this context, genome editing had been used to knock out CD52, allowing CAR-T cells to resist alemtuzumab, facilitating sustained lymphodepletion [23]. Other clinical studies also implemented this strategy (drug termed ALLO-647) to prolong lymphodepletion [39,40]; one trial highlighted that most responses were seen with a triple combination of fludarabine, cyclophosphamide and ALLO-647 (compared to just cyclophosphamide and ALLO-647), but use of ALLO-647 came with an anticipated risk of viral reactivation, especially CMV, which affected 14/43 participants [40]. In addition to recognised enhanced CMV risk [109], alemtuzumab is associated with significant additional toxicities [107], with frequent >grade 3 adverse events [33], a finding that may prove unacceptable in the treatment of non-malignant disease. However, in a clinical study of CNTY-101, a CAR-NK drug enhanced with gene edits, additional CAR cell infusions were given to two participants, without any additional lymphodepletion. In these patients, CAR-NK persistence was not negatively affected, with the baseline lymphodepletion alongside ‘alloevasion’ gene edits sufficient to prevent rejection [49]. Although only tested in a very small cohort, this is a promising result from an efficacy and cost-effectiveness standpoint. It should also be noted that the rheumatology community is broadly unfamiliar with the clinical delivery of lymphodepletion, requiring multidisciplinary implementation with haematology colleagues and training of medical and nursing personnel. Given the associated financial, staffing, and clinical delivery challenges, research into minimising the intensity of lymphodepletion required for allogeneic CAR-T treatment for autoimmunity should be a priority.

### 3.7. Enhancing Efficacy and Persistence

Limited persistence is a key challenge to the clinical implementation of CAR-based therapies, especially with CAR-NK cells [47,110]. Proposed options to enhance persistence include multiple dosing regimens, which, although effective preclinically [105], require additional costs, resources and patient contact that make them a clinically undesirable option. Hence, investing in strategies that improve CAR-T efficacy and persistence may be a long-term, more appealing option.

Alternative strategies explored often involved cytokine supplementation, most commonly IL-15 for CAR-NK, due to its role in enhancing NK expansion and persistence [98]. While numerous CAR-NKs were engineered to secrete IL-15 [48,88,98,111,112,113,114], others investigated co-expressing IL-15 linked to its receptor, IL-15/IL-15R(α) [90,94,104,105,115]. In the case of CAR-19 NK-92 cells, armouring with IL-15/IL-15Rα led to enhanced in vitro proliferation (in the absence of IL-2) and in vivo tumour control compared to soluble IL-15 [115]. Another cytokine-based strategy involved the addition of IL-21 alongside IL-2 to the CAR-NK expansion medium. This led to a higher yield of preferred phenotype CD62L^+^ cells, with enhanced cytotoxicity in preclinical studies [116]. While IL-15 strategies have already been implemented in clinical studies of CAR-NK products [46,49,50,52], it remains important to thoroughly investigate the safety of supplementary cytokine approaches in such clinical candidates, since IL-15 may increase the risk of CRS (albeit apparently more so with conventional CAR-T rather than CAR-NK) [88], as well as having CD8^+^ T cell-activating properties that may accelerate allorejection [117]. In addition, cytokine armouring may be associated with hyperleukocytosis, as was recently reported [118].

Another approach to improving efficacy, specifically of CAR-NK cells, is pretreatment with proteasome inhibitors. Proteasome inhibitors (PI) aim to ‘synergistically’ enhance NK cell therapy efficacy by altering the proteome of the target malignant cells, upregulating activating stress receptors and reducing the expression of inhibitory HLA-I molecules [119]. This was demonstrated preclinically, where pretreatment with the PI bortezomib was well-tolerated and enhanced the efficacy of CAR-NK cells against AML with significantly prolonged xenograft survival. By contrast, healthy cells, particularly HSPCs (haematopoietic stem and progenitor cells), were much less susceptible to PI effects, with 10- to 100-fold higher IC50 values necessary to induce their killing [119]. Although the prior study focused on bortezomib and CAR-NK for leukaemia, PIs have previously shown potential for autoimmune disease therapy [120]. Illustrating this, successful patient outcomes were reported with bortezomib for refractory SLE, accompanied by circulating plasmablast depletion [121]. Although further exploration and management of side effects would be necessary [120], evidence of autoimmune cell targeting by PIs suggests the applicable potential for combination with allogeneic, autoimmune CAR-T therapies.

### 3.8. Manufacture of Allogeneic CAR-Engineered Products

Most of the genome-edited trials used apheresis as a source of starting material for the manufacture of CAR-engineered products. Alternative approaches included ‘induced pluripotent stem cell’ (iPSC)-derived CAR-T/ NK [34]. This represents a renewable, standardised source, facilitating large-scale batch production and off-the-shelf availability. Another preclinical trial used ‘human placental T cells,’ which maintain a less-differentiated phenotype when expanded, favouring immune tolerance and proliferative potential [122]. Furthermore, ‘umbilical cord blood’ (UCB)-derived T cells, specifically the CD26L^+^ memory subset, were also used, showing strong expansion and functional potential, and providing ready access to a diverse range of HLA haplotypes, which could be beneficial in generating allogeneic products with reduced immunogenicity for use across a broad population [123]. Umbilical cord blood has similarly been used as an NK cell source in several studies [47,98,124]. These methods all provide options for enhanced allogeneic development.

A downside of using ‘unconventional’ lymphocytes is that they generally only make up a small fraction of circulating cells [70], creating potential challenges for the isolation of sufficient quantities [8] from starting material, coupled with the relatively limited expansion capabilities of some subsets such as NK cells [125]. One strategy commonly implemented to overcome this challenge entails manufacturing CAR-NK from established NK lines, such as NK-92 [125]. These cells are already ‘FDA-approved’ as a cell therapy host for the treatment of certain malignancies, and have the further advantage that they can be subject to efficient, large-scale expansion [126]. An alternative immortalised NK cell line that has been investigated is KHYG-1 [127], which has shown significantly greater cytotoxicity than NK-92 in prior preclinical studies [128].

An alternative emerging method to manufacture unconventional CAR cells such as CAR-NK involves the use of regenerative stem cells as the starting material. Firstly, engineering iPSCs allows for the controlled development of a clonal master cell bank, from which homogenous CAR products can be derived, for uniform, scalable production [46]. In addition, while primary NK cells can be difficult to genetically modify, the genome editing of source stem cells can overcome this limitation. Moreover, the incorporation of multiple genetic modifications in embryonic stem cells (ESCs) did not impair their differentiation and enabled the production of hypoimmunogenic CAR-NK cells, providing a reportedly much more ’cost-effective’ solution to mature NK cell transduction [92]. For sustainable production, the CAR-NK product would ideally be frozen to be stored and thawed for later use [46]. However, this could present a limitation, with cryopreserved NK having shown reduced motility and function compared to fresh cells [129]. Some evidence gathered in this review partially refuted this viewpoint, with frozen ‘FLT3 CAR_sIL-15 NK cells’ showing no significant phenotypic or functional difference to fresh cells [114]. Nonetheless, strategies to overcome this potential issue, such as post-thaw cytokine supplementation (e.g., IL-2) [130], to reactivate NK cells, should be further investigated for CAR-NK clinical manufacture.

A key commercial benefit of allogeneic over autologous CAR products is the removal of the need for the individualised collection of starting material (e.g., leukapheresis) and batch manufacture. This saves on time, the need for an invasive procedure and on costs of consumables and manufacturing infrastructure through the production of multiple doses per batch [6]. Illustrating this, each TyU19 batch normally produced over 100 patient doses [18]. Notably, one study utilised ‘leftover PBMC product’ from platelet donors as one source of cells, suggesting a potential way to repurpose donor product to save the costs of primary leukapheresis [73]. However, in principle, costs could be further reduced by removing the need for leukapheresis altogether, instead using whole blood as a starting material. Recent clinical trials have shown that clinically viable CAR-T cells can be produced from patient-derived blood samples as small as 40 mL [131].

The availability of pre-manufactured banks of allogeneic CAR-T/ NK, targeting varying specific biomolecules, could also facilitate the implementation of combination therapy approaches [6], which may aid treatment of complex autoimmune diseases [2]. This could provide scope for a degree of therapy personalisation, but more cost-effectively than autologous approaches. Moreover, repurposing pre-existing approved CAR-T products from malignancy to autoimmunity could also reduce costs associated with design and preclinical testing [14].

### 3.9. CAR Design and Transduction

The optimisation of CAR design is a further consideration for enhancing the efficacy of allogeneic CAR therapy. For example, ‘Dual-Targeting’ CARs, with ‘bi-specific’ molecular targeting, present benefits including a potentially enhanced antitumour effect [132], and the ability to overcome ‘antigen escape’ limitations [21,33]. Another multiple targeting approach is through the co-expression of additional receptors in the CAR product, such as a highly non-cleavable CD16 molecule (hnCD16). This facilitates the induction of antibody-dependent cellular cytotoxicity, for example, when rituximab (a CD20-specific antibody) is administered together with CAR NK cells. This approach also benefits from the harnessing of inherent NK cytotoxic mechanisms and CAR-directed targeting, creating a ‘triple-targeting’ drug product [104]. Similarly, hnCD16 was implemented in a BCMA-CAR-NK, enabling its use to target multiple myeloma together with the anti-CD38 antibody daratumumab. This approach enhanced tumour control, with maximal, sustained, in vivo efficacy seen in combination with a γ-secretase inhibitor (GSI), which elevates BCMA surface expression on target cells [105].

Furthermore, modifying the internal CAR architecture can profoundly impact functioning. For example, the use of a ‘specifically engineered’ NK receptor-based CD19-CAR, with DAP10, 2B4 and CD3ζ signalling, CD8α Hinge (H), and CD28 Transmembrane (TM) domains, demonstrated improved, potent cytotoxicity compared to a conventional T cell-optimised CAR (CD28 and CD3ζ signalling domains) as a CAR-NK therapy [133]. However, a contrasting study found that CD28 was a superior intracellular domain to 2B4 in a BCMA-CAR-NK [113]. Although this comparison was only undertaken in vitro, it suggests potential for further investigation and clinical comparison of different intracellular CAR constructs for various specific target antigens. Other manufacturing developments include the use of nanobody (VHH) derived CARs, which employ a small-sized targeting moiety with low immunogenicity but high binding affinity for more simple, feasible CAR construction [111]. Another study investigated the use of a variable lymphocyte receptor (VLR) as a CAR targeting moiety. However, when compared against a single-chain variable fragment (scFV) immunoglobulin-based CAR, this approach proved less effective in vivo, with a significant reduction in survival [134].

An alternative approach to achieve precisely targeted CAR transgene delivery involves the use of adeno-associated viruses (AAV) to specifically deliver CAR transgenes to the *TRAC* locus, which is simultaneously disrupted. This could theoretically provide more physiological control over CAR expression (employing the endogenous TRAC promoter). Moreover, this may reduce the risk of insertional mutagenesis associated with integrating retroviral or lentiviral vectors, with AAV suggested as a particularly effective and safe (low-level non-homologous capture) vector [135]. Preclinically, AAV was further shown to be a precise [136], high-efficiency [56] option, suggesting scope for clinical investigation as a specific, safe transduction tool. Non-viral vectors represent another option, with both ‘piggyBAC‘ [66,92] and ‘Sleeping Beauty’ [137,138] transposons showing preclinical success. Despite showing a favourable safety profile in these trials [66], piggyBAC has been linked to the onset of ‘donor-derived T cell lymphoma’ in two CAR-T recipients, a finding attributed to manufacturing rather than vector-related issues [139]. Although the Sleeping Beauty transposon system is believed to have a low risk of genomic instability, more investigation is needed into the insertional oncogenesis risk associated with this vector system [137]. On the other hand, CAR-encoding linear DNA [140] and mRNA [93] ‘electroporation’ transduction are non-viral methods [140], with the latter removing the risk of ’transgene-mutation’, since mRNA does not integrate into the host genome [93]. Building on this, there is also considerable interest in the use of more advanced transport mechanisms/vectors to drive ’negatively charged mRNA’ into cells, such as lipid nanoparticles [93].

### 3.10. Genome-Edited vs. Non-Genome-Edited Allogeneic CAR Therapies

Both conventional genome-edited CAR-T cells and ‘non-genome-edited’ CAR products have their own, contrasting advantages and limitations. Some preclinical studies included carried out experiments directly comparing conventional αβ to unconventional-source CAR cells, providing interesting comparisons. One notable head-to-head comparison of CD19-CAR-transduced cells found that although CAR-NK achieved comparable or even higher cytolytic activity against in vitro cell lines, CAR-T achieved significantly higher levels of ‘CAR-mediated killing’ and IFN-γ effector cytokine production. Further in vivo studies then demonstrated that autologous CAR-T cells significantly outperformed allogeneic CAR-NK cells, suggesting that allogeneic CAR-NK products may require higher/multiple doses or further modifications to improve clinical efficacy. Regarding repeated dosing of CAR-NK, it should be noted that this enabled the attainment of tumour control in one model, but not another [141]. On the other hand, a clinical study of ADI-001, a γδ CAR T product, found that in four lymphoma patients who had previously relapsed after autologous anti-CD19 therapy, all achieved CR after ADI-001 [51]. Another preclinical study supported this advantage, suggesting that γδ cells carry an advantage over αβ cells, especially in ’high-stress’ environments, with CAR-γδ showing greater, longer-lasting cytotoxicity at lower E:T ratios [96]. Furthermore, with the preferential ‘tissue-homing’ characteristics of some γδ T cell subsets [142], derived CAR therapies may represent particularly potent therapeutic options for certain autoimmune diseases.

### 3.11. Focus on Autoimmune Disease Application

Across the clinical studies included in this review, there were promising rates of CAR-T expansion and complete responses. Arguably, the strongest clinical proof of concept for the use of this approach in autoimmune disease was the profound responses observed in all three subjects treated with the allogeneic TyU19 CAR-T cell product [18]. Other notable findings were marked symptom score increases, a reduction in myositis inflammation, and even reversal of skin, lung, and heart fibrosis (systemic sclerosis); such tissue infiltration is not observed with B cell-depleting monoclonal antibodies, e.g., rituximab [2]. Similarly, the reversal of fibrosis was demonstrated in one subject with this disease following treatment with iPSC-derived CAR NK cells [43]. These results demonstrate clear potential for such CAR therapies to not only improve functioning/quality of life in patients, but also to reverse severe fibrosis, previously thought to be irreversible. Furthermore, not only was B cell depletion achieved, but patients also showed the complete loss of detectable autoantibody production, with another still showing clinical improvement despite only temporary B cell depletion [18]. Similar successful results have been presented in conference abstracts of allogeneic CAR studies, with one case series showing 66.7% SLE remission at 12 months [44], and another SLE study demonstrating sustained clinical responses, significant serum dsDNA (autoantibody) reduction and 3-month remissions with little or even no steroid supplementation [16].

Circulating TyU19 levels gradually decreased over time, accompanied by deep B cell depletion for 2–3 months. However, clinical remission was sustained at 6 months, indicating that shorter-term depletion was sufficient to achieve immune reset. Similar findings were reported in lupus patients with received the allogeneic “STAR CAR”, YTS109. While only two subjects in this study showed complete remission at 3 months, all five met the SLE responder index 4 (SRI-4) criteria, the primary efficacy endpoint, through to month 6 [19]. It remains to be seen how long clinical benefit will persist for and whether some subjects have indeed been cured of their disease.

The evaluation of a hypoimmune CAR-T product in clinical studies that implemented CD47 overexpression, including one patient with extra-renal lupus, has demonstrated promising allorejection evasion capability [143]. Another potential benefit over autologous CAR-T is the fact that residual CAR-T immunogenicity should eventually lead to immune rejection, avoiding the theoretical risk of long-term transformation [18]. Nonetheless, hypoimmunity strategies such as removal of all HLA molecules could conceivably lead to complete immune escape, in which case incorporating previously described ‘off-switches’ would facilitate subsequent elimination of infused cells.

All subjects who received allogeneic CAR-engineered cells for the treatment of autoimmune disease were heavily pre-treated, with active disease at the time of infusion. One subject had failed treatment with B cell depleting antibodies, raising the possibility that allogeneic CAR-T offers a more potent therapeutic approach [18]. A preclinical study of a CAR-NK product supported this claim, with ‘daratumumab resistant’ bone marrow cells efficiently lysed by the allogeneic CAR product [127]. Another disadvantage of monoclonal antibodies is the need for frequent, typically monthly administration, which a single-dose CAR-T product would obviate [2]. Overall, this suggests that allogeneic CAR-T therapies could overcome current limitations in the treatment of refractory B cell autoimmune disease [18].

Other preclinical studies of autoimmune-focused CAR products provide further proof of effect to support ongoing development. KYV-201, an allogeneic CRISPR-edited CAR-T drug, has achieved promising B cell depletion and in vivo disease control in preclinical autoimmune investigations [14], while CNTY-101, a gene-edit-enhanced CAR-NK cell product, elicited effective in vitro depletion of B cells from SLE patients [48]. Promisingly, the latter is already being implemented in a clinical study for CD19-positive B cell malignancies, with a reassuring safety profile [49], as well as further supporting the repurposing of pre-existing malignancy CARs without modification for autoimmune therapy.

### 3.12. Limitations of the Study

A key limitation of this review was the small number of published results on the clinical use of allogeneic CAR-T for the treatment of autoimmune disease, with only 5/107 such studies identified. Accordingly, most included studies were focused on blood malignancies. However, these publications are still highly relevant, since both strategies similarly aim to deplete B cells that express common lineage-specific target molecules, e.g., CD19 [2]. In addition, technologies used to design and express CARs, undertake genome editing and improve safety can be extrapolated from cancer to autoimmune therapy. Importantly, a key difference is that while treating cancer relies on sustained CAR-T cell persistence, sometimes bridging patients to an allogeneic stem cell transplant [27], an ‘immune reset’ for autoimmune treatment relies on a deep but shorter duration of B cell depletion [18]. Considering that persistence has been a key limiting factor for the success of CAR-based treatments for malignant disease, this may favour the use of these therapies in patients with autoimmune disease.

Other limitations include small sample sizes, with most clinical studies being in an early phase, with few subjects reported. Furthermore, all studies in autoimmunity presented very limited follow-up, meaning that later updates are eagerly awaited [18]. The limited quantity of clinical studies was a particular issue for unconventional, non-genome-edited therapies. Hence, to widen the breadth of reviewable data, preclinical studies were included, conscious of the many limitations of this data source. Similarly, many sources were conference abstracts or non-peer-reviewed reports, incurring a higher risk of bias. Such condensed abstracts also sometimes lacked specific clinical outcome data, such as duration of cytopenia.

There was also limited scope for a fair functional comparison between genome-edited and non-genome-edited strategies, since many non-genome-edited CAR products were compared against unedited αβ CAR-T cells, which would be expected to be more alloreactive and ineffective.

However, allogeneic CAR-T therapies are currently being investigated in several upcoming or ongoing clinical trials for a wide variety of autoimmune diseases (Table 3). Overall, this emphasises that despite limited numbers of published findings, this review focuses on a highly relevant, evolving and exciting area of interest.

## 4. Clinical Summary and Conclusions

Autologous CAR-T cells have achieved the durable remission of intractable autoimmune disorders owing to the profound depletion of circulating and tissue-resident B cells, followed by immune reset. However, these therapies are poorly scalable since, ultimately, a single batch of drug product only treats one patient. Allogeneic CAR-T sources provide a more commercially viable, scalable manufacturing alternative to autologous methods, which have predominated to date. Success requires that immunological barriers to safety (e.g., GVHD) and rejection can be addressed. Although clinical data are very limited, these therapies have demonstrated a proof of concept for positive clinical impact, with promising efficacy and acceptable safety outcomes. These early data clearly demonstrate its potential in an autoimmune context, with a variety of upcoming trials set to further expand this area of research. Both genome-edited and non-genome-edited allogeneic CAR products have shown promising ability to overcome alloreactivity barriers, with strong functional and safety profiles. Furthermore, combining approaches, for example, by genome-edit enhancement of unconventional CAR-T cells, could incorporate the individual benefits of each strategy to produce inherently cytotoxic, rejection-resistant therapies. Extrapolation from the clinical data generated when allogeneic CAR-T cells have been used to treat malignant disease highlights that these drugs may achieve less durable responses when compared to autologous products. Nonetheless, only transient B cell depletion has resulted in immune reset in the context of autoimmunity, meaning that the bar for success in autoimmunity may be lower than in cancer.

It should also be noted that the use of allogeneic products will impose many additional regulatory considerations for the deployment of these drugs. Guidance for industry on the development of human gene therapy products incorporating human genome editing (https://www.fda.gov/regulatory-information/search-fda-guidance-documents/human-gene-therapy-products-incorporating-human-genome-editing, accessed 6 November 2025) and allogeneic cell products (https://www.fda.gov/regulatory-information/search-fda-guidance-documents/safety-testing-human-allogeneic-cells-expanded-use-cell-based-medical-products, accessed 7 November 2025) has recently been produced by the US Food and Drug Administration.

In conclusion, these results, along with developing strategies to improve safety and scale and reduce the cost of manufacture, strongly suggest that allogeneic CAR-T therapies, both genome- and non-genome-edited, have strong potential to be commercialised as an autoimmune disease therapy.

## Figures and Tables

**Table 3:** Ongoing and upcoming clinical trials of allogeneic CAR-T for autoimmune disease.

ClinicalTrials.gov ID	CAR-T	Conditions
NCT05859997	BRL-301	SLE, Sjogren’s Syndrome, Systemic Sclerosis, Inflammatory Myopathy, ANCA-Associated Systemic Vasculitis, Antiphospholipid Syndrome
NCT06978738	CD19/BCMA UCAR	Inflammatory Myopathy, Autoimmune Haemolytic Anaemia, SLE, Systemic Sclerosis, ANCA-Associated Systemic Vasculitis, IgG4-RD, Myasthenia Gravis
NCT05988216	BRL-301	SLE
NCT06886919	IC19	Refractory SLE
NCT06939166	CD19/BCMA UCAR	Autoimmune Encephalitis, Multiple Sclerosis, Chronic Inflammatory Demyelinating Polyradiculoneuropathy, Myasthenia Gravis, Neuromyelitis Optica Spectrum Disorders
NCT06828042	CD19 CAR-γδT	Inflammatory Myopathies, SLE, Systemic Sclerosis, ANCA-Associated Systemic Vasculitis, Antiphospholipid Syndrome, Sjogren Syndrome
NCT06485232	Universal BCMA + CD19 CAR-T	Neuromyelitis Optica Spectrum Disorders, Myasthenia Gravis, Multiple Sclerosis, Chronic Inflammatory Demyelinating Polyradiculoneuropathy
NCT07085104	ALLO-329	Idiopathic Inflammatory Myopathy, Lupus Nephritis, SLE, Systemic Sclerosis
NCT06941129	CD19 + BCMA UCAR	SLE, Systemic Sclerosis, Inflammatory Myopathy, ANCA-Associated Systemic Vasculitis
NCT06920433	CD19/BCMA UCAR	SLE
NCT07115745	BMS-986515	Refractory Autoimmune Disease
NCT07155369	CD19/BCMA UCAR	SLE, Systemic Sclerosis
NCT06946485	CHT101	SLE
NCT06821659	UWD-CD19	Inflammatory Myopathies, SLE, Systemic Sclerosis, ANCA Associated Systemic Vasculitis, Sjogren Syndrome
NCT06691152	CD19 UCAR	SLE
NCT06633042	BCMA Universal CAR-T	AQP4 Antibody Positive Neuromyelitis Optica Spectrum Disease
NCT06340490	RJMty19	SLE
NCT06106893	CD19 γδ T	SLE
NCT06212154	ThisCART19A	Autoimmune Haemolytic Anaemia
NCT06752876	CB-010	SLE, Lupus Nephritis
NCT06980597	OL-108	Idiopathic Inflammatory Myositis, SLE, Systemic Sclerosis, ANCA-Associated Systemic Vasculitis
NCT06294236	SC291	SLE, ANCA-Associated Systemic Vasculitis, Granulomatous Polyangiitis, Microscopic Polyangiitis
NCT06733610	Universal anti-CD19/BCMA CAR T	Autoimmune Haemolytic Anaemia
NCT06983964	CD19 CART	Autoimmune Diseases
NCT06920446	CD19/BCMA UCAR	Autoimmune Haemolytic Anaemia
NCT07105735	RN1201	Autoimmune Disease
NCT07129642	Allogeneic Anti-CD19 CAR-T	Graves’ Disease
NCT07142161	RD13-02	Type 1 Diabetes
NCT06375993	ADI-001	Lupus Nephritis, SLE, Systemic Sclerosis, ANCA-Associated Systemic Vasculitis, Idiopathic Inflammatory Myopathies, Stiff Person Syndrome
NCT06925542	CTX112	Lupus Nephritis, SLE, Systemic Sclerosis, Idiopathic Inflammatory Myopathies
NCT07031713	CT1192	SLE
NCT06933563	CD19/BCMA UCAR	Myasthenia Gravis
NCT07072247	RN1201	Immune-mediated Platelet Transfusion Refractoriness, Immune Thrombocytopenia
NCT06986018	RD06-04	ANCA-Associated Systemic Vasculitis, Idiopathic Inflammatory Myopathies
NCT06308978	FT819	Idiopathic Inflammatory Myositis, SLE, Systemic Sclerosis, ANCA-Associated Systemic Vasculitis
NCT06686524	Anti-CD19 UCAR-T	Refractory Juvenile Dermatomyositis
NCT06681337	BCMA + CD19 CAR-T	Lupus Nephritis
NCT07100873	ADI-001	Rheumatoid Arthritis
NCT07033299	CT1192	ANCA-Associated Systemic Vasculitis

Trials were retrieved from ‘clinicaltrials.gov’, entering the same Boolean style search terms used for the autoimmune database search, but without either of the genome-edited or non-genome-edited method-distinguishing terms, were entered into the relevant search categories on the search page, to generate 40 results as of 7 September 2025. One of the trials retrieved in the search (NCT06429800) was excluded from this table due to being withdrawn. The other 39 trials have been recorded in the table, showing their trial ID, CAR-T product and autoimmune conditions being investigated.

## Data Availability

No new data were created in this review.

## Supplementary Materials

The following supporting information can be downloaded at: https://www.mdpi.com/article/10.3390/biology14121790/s1, Search methods, Figure S1: Review population, intervention, comparison and outcomes (PICO) criteria. These represent the key components extracted from the studies included in this review. (1) and (2) are used to refer to the two separate review sections with different intervention focuses; Figure S2: Inclusion and Exclusion Criteria. The key points specified in the table were guided by the ‘common inclusion/exclusion criteria’ categories specified in the University of Melbourne Library Guide (https://unimelb.libguides.com/sysrev/inclusion-exclusion-criteria, accessed on 24 December 2024). (1) and (2) are used to refer to the two separate review sections when they differ in inclusion/exclusion criteria. The third search for autoimmune disease included identical inclusion and exclusion criteria for clinical studies in autoimmunity only; Figure S3: Database search terms. Two database search inputs focused on autoimmune disease and cancer were formed using key search terms, linked by Boolean Operators. Each was inputted into PubMed, EMBASE (Ovid) and Web of Science Core Collection. Each search was split into 4 categories of search terms, namely allogeneic, CAR-T, gene edited, and condition being targeted, to best organise the search input. A variety of search term synonyms for each category and/or a list of examples was used to maximise the number of studies identified. The two search terms, γδ and Epstein–Barr virus-specific cytotoxic T cells, were not included in the EMBASE search only, due to an unrecognised syntax error; Supplementary Table S1: Preclinical studies using Genome-Edited CAR-T cells; Supplementary Table S2: Preclinical studies using Non-Genome-Edited CAR cells. Refs. [144,145,146,147,148,149,150,151,152,153,154,155,156,157,158,159,160,161,162,163] are cited in SM files.
