# Supplementary material for: Prospects for Development and Commercialisation of Allogeneic CAR-Based Therapies for Autoimmune Disease
Source: Biology (Basel). 2025 Dec 15;14(12):1790. doi: 10.3390/biology14121790 (PMC12730949; doi:10.3390/biology14121790)
Supplement: Supplementary file 1 [file biology-14-01790-s001.zip › biology-3939702-supplementary.pdf]

Supplementary Materials

Search Methods

This structured review of allogeneic CAR-T therapies for autoimmune disease was split and conducted in three stages. The first, original review focussing on, 'genome-edited' studies, and then the second, extended review focusing on 'non-genome edited' studies, to expand the review focus. The original data search was conducted in November 2024, with the second search in March 2025. At the time of the second search, the original search was repeated and newly published studies added to provide a comprehensive, up-to-date review with continuity of data across both sections. Finally, a third search of clinical studies of allogeneic CAR-T treatment of autoimmune disease was conducted in November 2025 to update this dataset as close to publication date as possible. The flow of studies through each screening stage is depicted in the PRISMA-style flow diagrams in the results section. The separate search and data collection processes were identical and have hence been grouped together in the upcoming methods description and figures.

The review questions and eligibility criteria were defined using a PICO [9] format (Figure S1 and Figure S2 respectively).

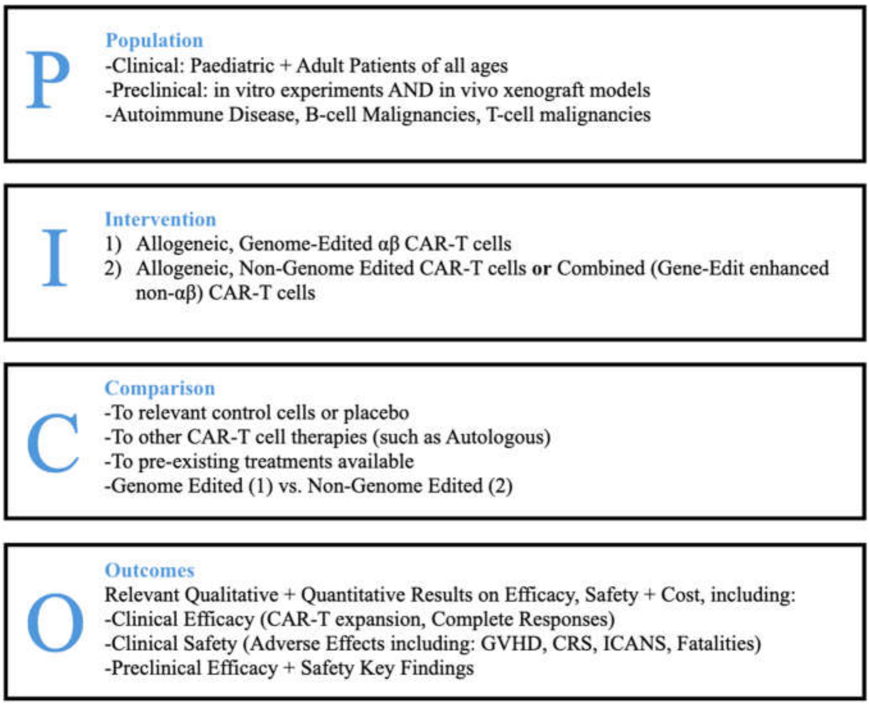

Figure S1. Review population, intervention, comparison and outcomes (PICO) criteria. These represent the key components extracted from the studies included in this review. 1) and 2) are used to refer to the two separate review sections with different intervention focuses.

Given the limited number of published allogeneic CAR studies in autoimmune disease, the search scope was expanded to include B and T cell haematological malignancy, to allow extrapolation of relevant insights. In the second 'non-genome edited' search, 'combination studies', were also included to maximise review evidence for analysis. Typically, these described cell sources such as NK and γδ cells that were enhanced with additional gene edits. For ease of understanding, it should be noted that the two review categories of 'genome-edited' and 'non-genome-edited' studies are often referred to in this review, but unless otherwise specified or highlighted, the latter also includes these

'combination' studies. Furthermore, 'gene-editing' refers to the utilisation of DNA editing technologies (e.g. CRISPR Cas-9, TALEN, Base-editing etc.) to disrupt the host cell genome, as opposed to CAR transduction or transfection, which evidently all CAR-T products included will have undergone.

Inclusion Criteria	Exclusion Criteria
Research Trials focussed on developing an Allogeneic, CAR-T cell therapy:<br>**1) Genome-edited**<br>**2) Non-genome edited or Combined**	Non-primary Research or Publications, including Reviews + Editorials<br>Articles/Abstracts with Insufficient, Irrelevant, or Non-evidence-based Data
Clinical and Preclinical Trials (with in vitro + in vivo data)	Novel Preclinical Studies Lacking In Vivo Experimental Data
Full Text Research Articles and Conference Abstracts	Studies focussing on Non-CAR Cellular Therapies
Peer Reviewed and Non-peer Reviewed Grey Literature	Studies of Autologous CAR-T Therapies
All ages, both Adult and Paediatric Clinical Trial Populations	**1) Non-genome edited source (NK, γδ, EBV-CTL etc.) CAR-T studies**
Interventions for Autoimmune Disease, B-cell + T-cell Haematological Malignancies	**2) Genome-edited αβ source CAR-T studies**
Quantitative + Qualitative Outcome Sata on Safety and Efficacy of the Intervention	Studies Focussing on T-regs or In Vivo CAR-T<br>Preliminary Trials focussed on a Generic Manufacturing Strategy without a Clear Disease Intervention Target or Developable CAR-T Cell Therapy
Any Geographic Location	
Any Study Setting	Interventions for Solid Tumour, Non-Haematological Malignancies or Non-specified other Conditions/Applications
Published any time, up until week of 10-17th March 2025	Earlier (or less sufficient/suitable data) Duplicate Publications of a Trial
	Preclinical Trials focussed on a Therapy which is now in Clinical Trials with Results Available
	Articles Not Available in the English Language
	Articles Not Retrievable for Free through King's College London Institution

Figure S2. Inclusion and Exclusion Criteria. The key points specified in the table were guided by the 'common inclusion/exclusion criteria' categories specified in the University of Melbourne Library Guide (https://unimelb.libguides.com/sysrev/inclusion-exclusion-criteria, accessed on 12/12/24). 1) and 2) are used to refer to the two separate review sections when they differ in inclusion/exclusion criteria. The third search for autoimmune disease included identical inclusion and exclusion criteria for clinical studies in autoimmunity only.

The searches were conducted across three databases: PubMed, EMBASE (Ovid) and Web of Science Core Collection. To manage the results, for each review stage, two separate searches were undertaken per database; one for autoimmune disease and another for haematological malignancies. Search terms were combined using Boolean Operators (Figure S3).

The list of autoimmune search terms was formulated using common diseases [2] and two less common conditions that a current clinical study is focussed on [18]. Similarly, the list of haematological malignancy terms was formulated to cover key B and T cell cancers [63].

Allogeneic AND CAR-T AND Method AND Condition:

Genome-Edited Autoimmune Search:

(allogeneic OR universal OR off-the-shelf OR donor OR allo) AND (CAR-T OR Chimeric Antigen Receptor T-Cells) AND (gene-edited OR genome-edited OR CRISPR OR TALEN OR Base-edited) AND (autoimmune disease OR autoimmune condition OR rheumatoid arthritis OR systemic lupus erythematosus OR type 1 diabetes OR pemphigus OR multiple sclerosis OR systemic sclerosis OR myositis)

Genome-Edited Malignancy Search:

(allogeneic OR universal OR off-the-shelf OR donor OR allo) AND (CAR-T OR Chimeric Antigen Receptor T-Cells) AND (gene-edited OR genome-edited OR CRISPR OR TALEN OR Base-edited) AND (B-cell malignancy OR T-cell malignancy OR leukaemia OR lymphoma OR myeloma)

Non-Genome-Edited Autoimmune Search:

(allogeneic OR universal OR off-the-shelf OR donor OR allo) AND (CAR-T OR Chimeric Antigen Receptor T-Cells) AND (non-gene-edited OR non-genome edited OR Natural Killer OR NK OR gamma-delta OR gd OR $\gamma\delta$ OR *Epstein–Barr virus-specific cytotoxic T-cells* OR EBV-CTL) AND (autoimmune disease OR autoimmune condition OR rheumatoid arthritis OR systemic lupus erythematosus OR type 1 diabetes OR pemphigus OR multiple sclerosis OR systemic sclerosis OR myositis)

Non-Genome-Edited Malignancy Search:

(allogeneic OR universal OR off-the-shelf OR donor OR allo) AND (CAR-T OR Chimeric Antigen Receptor T-Cells) AND (non-gene-edited OR non-genome edited OR Natural Killer OR NK OR gamma-delta OR gd OR $\gamma\delta$ OR *Epstein–Barr virus-specific cytotoxic T-cells* OR EBV-CTL) AND (B-cell malignancy OR T-cell malignancy OR leukaemia OR lymphoma OR myeloma)

Figure S3. Database search terms. Two database search inputs focused on autoimmune disease and cancer were formed using key search terms, linked by Boolean Operators. Each was inputted into PubMed, EMBASE (Ovid) and Web of Science Core Collection. Each search was split into 4 categories of search terms, namely allogeneic, CAR-T, gene edited, and condition being targeted, to best organise the search input. A variety of search term synonyms for each category and/or list of examples was used to maximise the number of studies identified. The two search terms in italics, $\gamma\delta$ and *Epstein–Barr virus-specific cytotoxic T-cells*, were not included in the EMBASE search only, due to unrecognised syntax error.

Studies retrieved were screened, aided by the platform 'Rayyan' [144]. Firstly, automated article duplicates were filtered and removed. Resource titles and abstracts were then initially screened to exclude incorrect publication types, interventions, or study focus. Remaining studies were then sought for retrieval before an in-depth screen of full texts was performed. Next, included studies underwent data extraction. Relevant key results and outcomes were recorded, then condensed and synthesised into four separate tables (clinical and pre-clinical trials, from genome-edited and non-genome edited review sections). Risk of bias and study quality concerns were qualitatively considered by the first author dusing screening, based on study design, completeness of data and publication type. Certain articles were excluded on grounds of inadequate bias risk (insufficient/vague data). Preclinical studies and non-peer reviewed grey literature (conference abstracts) were included, maximising the breadth of research analysed. However, these articles carry higher risk of bias, so most weighting was given to peer-reviewed, full-text studies. Preclinical studies were only included if they had clearly evidenced, in vivo data. One exception to this was an early in vitro autoimmune-focussed CAR-NK study, which was included because the same CAR product had already been utilised and included in a clinical study for haematological malignancy [48]. References were formatted using 'Endnote' reference manager (version 21). This review was informed by PRISMA 2020 reporting principles where applicable [145]. Generative artificial intelligence was not used in the preparation of this paper.

Supplementary Table S1. Preclinical studies using Genome-Edited CAR-T cells.

Description	CAR-T production	Study design	Key Findings	Ref.
KYV-201: anti-CD19 CAR-T for autoimmune disease	Allogeneic T cells were transduced using an undisclosed method. CRISPR Cas9 was used to knockout *TCR, B2M* and one other undisclosed gene.	In vitro and xenograft models of autoimmune disease	KYV-201 controlled a NALM6 B-cell leukaemia xenograft with similar efficiency to unedited anti-CD19 CAR-T controls. This was accompanied by donor B cell elimination and KYV-201 CAR T expansion. There was no evidence of T or NK cell-mediated rejection of KYV-201 in vitro. When KYV-201 was evaluated at clinical scale, high transduction efficiency and on-target editing were achieved with minimal off target effects, translocations, or chromosomal rearrangements. There was no evidence of uncontrolled proliferation in absence of cytokines.	[14]
7CAR8: an anti-CD7 CAR-T for T-ALL	Healthy donor T cells were transduced using a lentiviral vector. Cytosine base editing was used to simultaneously edit *B2M, TCRα, PD1* and *CD7* loci.	In vitro and xenograft models of T-ALL, including PDX	7CAR8 treated mice all survived to end of trial and those that had received the highest dose had no detectable disease. *PD1* knockout further prolonged median survival in PDX mice compared to WT controls (21w vs 15.9w) without adverse effects.<br>Cytosine base editing caused no increase in translocations compared to control cells in contrast to CRISPR Cas 9 edited cells.	[68]
UCART7: a universal anti-CD7 CAR-T for r/r T-ALL + T-NHL	Allogeneic T cells were transduced using a lentiviral vector. CRISPR Cas 9 was used to knock out *TRAC* and *CD7* loci.	In vitro and xenograft models of T-ALL including PDX	UCART7 achieved significantly enhanced control of T-ALL PDX compared to controls. T cell fratricide was significantly mitigated with CD7 deletion. There was no evidence of xenogeneic GVHD in mice. A degree of on- and off-target nuclease activity was observed, but without evidence of gene arrangements.	[146]
SB-CAR/TCR KO: an anti-CD19 CAR-T for $CD19^+$ lymphoid malignancy	Purified human T cells were transduced with the Sleeping Beauty (SB) transposon system. CRISPR Cas9 was used to knock out the *TRAC* locus.	In vitro and in vivo models of $CD19^+$ malignancy	Sleeping Beauty CD19-28z.CAR T cells with TCR knock out showed equivalent tumour suppression and survival prolongation when compared to non TCR knock out CAR-T controls and significant survival prolongation compared to CAR-T controls. TCR knockout led to protection against GVHD. There was a low rate of Sleeping Beauty transposition at potential oncogenic sites.	[137]
HIP CD19 CAR T: a Hypoimmune anti-CD19 CAR-T for $CD19^+$ malignancy	Human T cells were transduced with a bicistronic lentiviral vector encoding for the CAR and CD47. CRISPR Cas 9 was used to knock out *B2M, CIITA* and *TRAC* loci.	In vitro and in vivo models of $CD19^+$ malignancy	HIP CD19 achieved target cell killing and trafficking to bone marrow and spleen equivalent to or better than control allogeneic CAR-T cells. Moreover, it did not cause GVHD in contrast to control allogeneic CAR-T cells. In immunocompetent mice, HIP CAR T achieved greater persistence and tumour control in contrast to non hypoimmune allogeneic control CAR-T cells.	[58]
TCR-KO-CAR-T: an anti-CD19 CAR-T for leukaemia	Healthy donor T cells were transduced with a retroviral vector. CRISPR Cas-9 was used to knock out *TCRβ*	In vitro and in vivo models of $CD19^+$ leukaemia	TCR-KO-CAR-T achieved rapid control of PDX tumours accompanied by markedly enhanced survival compared to untransduced controls. However, in a NALM6 B-ALL xenograft model TCR-KO-CAR-T cells demonstrated reduced persistence and efficacy compared to TCR^+ cells. Nonetheless, TCR-KO-CAR-T were associated with reduced occurrence of GVHD compared to TCR intact control cells.	[54]

BCMA/CD47-directed UCAR-T: a bispecific BCMA/CD47 CAR-T for multiple myeloma	Healthy PBMCs were subjected to lentiviral transduction. CRISPR Cas-9 was used to edit the *TRAC* and *B2M* loci.	In vitro and in vivo models of multiple myeloma	Bispecific UCAR-T achieved greater cytotoxicity, antitumour effects and survival compared to monospecific equivalents and CD47 controls. No signs of GVHD were noted.	[132]
CAR-Vβ2 T: an anti-TCR-Vβ2 CAR-T therapy for (Vβ2⁺) T cell malignancy	Healthy donor T cells were transduced with a lentiviral or AAV vector. CRISPR Cas-9 was used to knock out of *TRAC, B2M* and *CIITA* loci.	In vitro and in vivo models of T cell malignancy	Mice with a peripheral T cell lymphoma PDX showed a significant reduction in disease burden compared to controls, accompanied by higher CAR-T persistence. Using a humanised CAR derivative, they showed similar levels of expansion and antitumour efficacy in vivo. Transduction with AAV achieved precise transgene delivery to the *TRAC* locus, accompanied by comparable safety and efficacy to lentiviral transduced cells.	[136]
HLA-I KO/TCR KO CAR-T: an anti-CD33 CAR-T for r/r AML	CRISPR Cas-9 was used to knock out *TRAC* and *B2M* loci. The Sleeping Beauty transposon/ transposase system was used to encode for the CAR.	In vitro and in vivo models of AML	Engineered knock out cells demonstrated slightly reduced proliferation compared to control CAR-T cells, but no significant difference between cytotoxicity and mouse survival rates were observed. There were similar levels of Sleeping Beauty transposon copies in both edited and non-edited CAR-T and no significant CRISPR off-target effects were seen. Large-scale manufactured CD33-CAR-TCR KO cells were equally functional.	[138]
U7CART: an anti-CD7 CAR-T for T-cell malignancy	Healthy donor PBMCs were transduced with a lentiviral vector. CRISPR-Cas9 was used to knock out the *TRAC* and *CD7* loci.	In vitro and in vivo models of T cell malignancy	U7CART achieved specific, on target killing of $CD7^{+}$ cells. When tested in vivo, tumour suppression and survival were significantly increased compared to controls. Knock out of CD7 was associated with a shift towards a $CD4^{+}$ phenotype. In CD19 targeted CAR-T, CD7 knock out was accompanied by a small increase in cytotoxic activity	[147]
TCRKO ARI-0001: an anti-CD19 CAR-T therapy for B cell lymphoma	Healthy donor PBMCs were transduced with a lentiviral vector. CRISPR Cas-9 was used to knock out the *TRAC* locus.	In vitro and in vivo models of B cell lymphoma	Efficacy of TCRKO ARI-0001 against established tumour and re-challenge was comparable to wild type control CAR-T cells despite a small reduction in proliferation and tissue abundance of the cells. Treatment with TCR knock out cells also resulted in a significant reduction in xenogeneic GVHD. Large on target deletions were detected in the *TRAC* locus, raising a potential safety risk.	[148]
UCART123: an anti-CD123 CAR-T for AML	Healthy donor PBMCs were transduced with a lentiviral vector which incorporated the RQR8 kill switch. TALEN editing was used to knock out the *TRAC* locus	In vitro and in vivo models of AML, including PDX models	UCART123 treatment resulted in complete elimination of AML PDX, accompanied by a significant survival prolongation and an absence of GVHD (unlike control mice). Relapses that were observed following low dose treatment were $CD123^{+}$ and responded to a second CAR-T cell infusion. Treatment also achieved selective elimination of leukaemia cells, sparing normal human bone marrow in mixed xenograft models. Rituximab was shown to effectively eliminate UCART123, validating the kill switch technology.	[149]
CB-011: a hypoimmune, anti-BCMA CAR-T for multiple myeloma	Healthy donor PBMCs were engineered using Cas12a RNPs complexed with chRDNA guides directed to *TRAC* and *B2M* loci. AAV-6 homology-directed repair (HDR) donor templates were used to knock	in vitro and in vivo models of multiple myeloma	Knock in cells achieved significant tumour reduction and prolonged xenograft survival without induction of GVHD, unlike control treated mice. Expression of the *B2M-HLA-E* polyprotein protected cells from NK cell mediated rejection. No detectable off-target editing was attributed to Cas12a editing, although a low balanced *TRAC*/*B2M* translocation frequency (0.02-0.05%) was noted.	[56]

	the CAR into the *TRAC* locus and a *B2M–HLA-E* fusion into the *B2M* locus.			
BC404-UCART: an anti-BCMA CAR-T, secreting anti-CD47 Nb-Fc fusion, for multiple myeloma	Healthy donor PBMCs were transduced with a lentiviral vector (incorporated anti-CD47 nanobody-Fc fusion). CRISPR Cas-9 was used to knock out the *TRAC* and *B2M* loci.	In vitro and in vivo models of multiple myeloma	CD47 blockade significantly enhanced CAR-T cell anti-tumour xenograft effect in vivo, leading to prolonged survival, with 100% survival at day 100. In vitro experiments showed anti-CD47 nanobody-Fc fusion secretion enhanced phagocytic capacity of macrophages targeting myeloma cells	[61]
TTCAR-19: a Terminal-*TRAC* anti-CD19 CAR-T for B cell malignancy	Transduction of T cells with a lentiviral vector that incorporated a Pol III promotor and single guide RNA sequence targeted to *TRAC* in the 3' LTR. CRISPR Cas-9 was used to knock out *TRAC*, coupling CAR expression and genome editing.	In vitro and in vivo models of B cell malignancy	Significant and rapid clearing of tumour cells was achieved by test and control CAR19 cell populations, compared to non-CAR controls. Tumour suppression was significantly prolonged compared to TCR^+ CAR19 cells. The TTCAR-19 group also showed the highest levels of CAR T cells compared to control groups. No overt toxicity was observed, although a minor increase in translocations was noted in the edited cells	[150]
UCART123: an anti-CD123 CAR-T for blastic plasmacytoid dendritic cell neoplasm (BPDCN)	Healthy donor PBMCs were transduced with a lentiviral vector that incorporated the RQR8 kill switch. TALEN technology was used to knock out the *TRAC* locus.	In vitro and in vivo models of BPDCN	UCART123 treatment resulted in significantly higher tumour control and survival of mice compared to controls. In one case (PDX3), relapse was attributed to antigen loss. By contrast, in a second PDX model (PDX2), all UCART123 mice died soon after CAR-T due to CRS. Earlier administration of CAR T cells in the PDX3 model extended survival with less severe CRS (due to lower tumour burden). Moreover,rituximab effectively induced cell clearance due to the RQR8 safety switch.	[151]
TUCAR-T + ETUCAR-T: anti-CD19 CAR-T for $CD19^+$ malignancy	Healthy donor PBMCs were transduced with a lentiviral vector. Triple edited TU-CAR-T were modified using CRISPR Cas-9 to knock out the *TRAC, B2M* and *CIITA* loci. Investigators also prepared HLA-E/B2M fusion expressing derivative (ETUCAR-T) and double edited (DU-CAR-T; *TRAC* and *B2M* edited) control cells.	In vitro and in vivo models of $CD19^+$ malignancy	TUCAR-T achieved comparable antitumour effect to control DUCAR-T and unedited CAR-T cells, but persistence of the cells was lower when compared to unedited CAR T cells. ETUCAR-T maintained antitumour efficacy compared to TUCAR-T, but in multiple infusion trial, despite initially better antitumour effect, it was associated with disease relapse and lower levels of CAR-T persistence compared to unedited CAR-T control cells that expressed HLA-E/ B2M fusion protein. Neither TUCAR-T or ETUCAR-T resulted in GVHD and both also resisted rejection by host immune cells.	[55]
Allogeneic BCMA CAR-T: an anti-BCMA CAR-T for multiple myeloma incorporating	Healthy donor PBMCs were transduced with a lentiviral vector (incorporated RQR8 safety switch). TALEN	In vitro and in vivo models of multiple myeloma.	Antitumour efficacy of this approach in vivo was replicated using large scale manufactured CAR-T. However, there was a high degree of disease relapse in xenograft models, with limited persistence and only short-term expansion. Co-administration of stabilised IL-7 + IL-15 (or AAV encoded cytokines) enhanced and	[152]

rituximab intra-CAR mimotope	technology was used to edit the *TRAC* and *CD52* loci.		prolonged this anti-tumour effect. Rituximab induced significant elimination of circulating BCMA CAR-T, acting via the RQR8 safety switch.	
P-BCMA-ALLO1: an anti-BCMA CAR-T for multiple myeloma	Donor T cells were transduced with the piggyBac Transposon system which incorporated the inducible caspase 9 kill switch. Cas-CLOVER was used to knock out the *TRBC* and *B2M* loci.	In vitro and in vivo models of multiple myeloma.	P-BCMA-ALLO1 T cells achieved strong in vivo antitumour efficacy and persistence, comparable to wild type CAR T cells. The cells were detectable in peripheral blood throughout, although at lower levels than control cells. Additionally, there was an insignificant trend for translocation increase compared to unedited cells, except for a small but significant increase in complex rearrangements of *TRBC*	[66]
TRAC:CAR-T cells: an anti-CD19 CAR-T for lymphoma	The CAR transgene was delivered to the *TRAC* locus of healthy donor T cells using TRC1-2 (*TRAC* specific endonuclease) and an AAV donor template.	In vitro and in vivo models of lymphoma	*TRAC* edited CD19 CAR T achieved significant expansion and tumour suppression in vivo. All mid and high dose treated mice survived along with most of the low dose mouse cohort. The AAV template proved to be effective, with high efficiency of integration, targeted *TRAC* locus integration, and low frequency of non-homologous AAV capture. It was therefore suggested that this approach lowers the risk of insertional mutagenesis and provides greater control over CAR expression.	[135]
UCART2: an anti-CD2 CAR-T for T-ALL + CTCL/T-NHL	Allogeneic T cells were transduced with a lentiviral vector. CRISPR Cas-9 was used to knock out the *TRAC* and *CD2* loci.	In vitro and in vivo models of T-ALL/T-NHL. Therapy was tested +/- rhIL-7-hyFc (long-acting IL-7 analogue)	UCART2 demonstrated in vitro efficacy against both T-ALL + CTCL cells. However, CD2 deletion compromised CAR function, owing to shortened survival in vivo. Although UCART2 was still effective alone, expansion, function, persistence and efficacy (indicated by survival of mice) was further enhanced in combination with rhIL-7-hyFc, which compensated for CD2 deficiency in T cells.	[15]
CD22 HIP CAR T: a hypoimmune anti-CD22 for CAR-T for r/r LBCL	CAR was co-delivered with CD47 to healthy donor T cells, by an undisclosed method. The *B2M, CIITA* and *TRAC* loci were edited using an undisclosed technology.	In vitro + in vivo models of B cell lymphoma	CD22 hypoimmune CAR T cells demonstrated significant in vivo antitumour efficacy compared to mock controls. They also achieved a significant survival prolongation and tumour control , comparable to control CD47-CD22 CAR-T in a systemic model. Hypoimmune cells evaded NK and macrophage recognition in vitro, unlike mock control cells (lacking additional CD47) that were rapidly killed.	[59]
BE-CAR33 +/or BE-CAR7: anti CD33/CD7 CAR-Ts for AML	Healthy donor lymphocytes, were transduced with a lentiviral vector. Base editing was used to knock out *CD7, CD52* and *TRBC.*	In vitro and in vivo models of AML, including PDXs	BE-CAR33 alone or in combination with BE-CAR-7 achieved enhanced control of AML PDX and increased survival, compared to CAR19 control or BE-CAR7 alone	[153]
UCARTCS1: an anti-CS1 (SLAMF7) CAR-T for multiple myeloma	Healthy donor T cells were transduced using a non-disclosed method. TALEN technology was used to knock out *TRAC* and *CS1* loci.	In vitro and in vivo models of multiple myeloma	UCARTCS1 achieved strong, dose dependent anti-tumour efficacy in vitro and in vivo. There was some lysis of non-malignant CS1^{+} cells in bone marrow samples, such as CD4^{+} & CD8^{+} T cells, B cells, NK cells, but this was less efficient than tumour cell lysis. Therapeutic activity was confirmed using samples from patients with low CS1 expression, high tumour burden or high Treg numbers.	[154]
Dual-transduced CD19 CAR x CD22 CAR–T: a hypoimmune, anti-	Healthy donor T cells were subjected to dual lentiviral transduction with single CAR encoding vectors which co-	In vitro and in vivo models of leukaemia/ lymphoma	Dual transduced CAR T cells achieved enhanced tumour control and survival compared to mock transduced cells. Efficacy was also greater than single CAR transduced control cells.	[60]

CD19/ CD22 CAR-T for leukaemia/ lymphoma	expressed CD47 overexpression. CRISPR-Cas12b was used to knock out *B2M*, *CIITA* and *TRAC* loci.			
anti-CD83 CAR-T: an anti-CD83 CAR-T for AML + GVHD reduction	The CAR gene was delivered to the *TRAC* locus in allogeneic T cells using an undisclosed method. CRISPR Cas-9 was used to knock out the *TRAC*, *B2M*, *CD83*, *ZC3H12A* and *TGFBR2* loci.	In vitro and in vivo models of AML and GVHD	CD83 is expressed on AML cells and immune cell mediators of GVHD. With the triple knock out of *CD83*, *ZC3H12A* (encodes Regnase-1) and *TGFBRII*, in vitro CAR T cell expansion was maintained, anti-leukaemic activity was enhanced and xenogeneic GVHD was prevented. By contrast, the same dose of CD83 single knock out CAR T cells exhibited delayed onset of GVHD. Anti-GVHD activity of CD83 single knock out T cells could also be enhanced by adding the CTLA4-Fc fusion, belatacept.	[155]
P-CD19 CAR-T: human placental cell-derived, anti-CD19 CAR-T for B cell malignancy	Human Placental Derived Allogeneic T cells were engineered by retroviral transduction. CRISPR Cas-9 was used to knock out the *TRAC* locus.	In vitro and in vivo models of $CD19^+$ malignancy	P-CD19 CAR-T achieved a significant tumour burden reduction and survival prolongation in vivo compared to the vehicle alone group). There were no signs of GVHD (fur loss/weight loss) although it should be noted that unedited human placenta-derived CAR-T did not induce GVHD in this model.	[122]
4KO-LLT1 CD38 UCAR-T: an anti-CD38 CAR-T for T-ALL, AML + lymphoma	Peripheral blood T cells were modified by lentiviral transduction with a vector that also encoded for LLT1. CRISPR Cas-9 was used to knock out the *TRAC*, *B2M*, *CIITA* and *CD38* loci.	In vitro and in vivo models of B + T cell malignancy	4KO-LLT1 CD38 UCAR-T demonstrated robust in vitro and in vivo anti-tumour activity. In an in vivo T-ALL model, 4KO-LLT1 CAR-T mice achieved the greatest expansion in peripheral blood, accompanied by more durable remission and survival, compared to all controls.	[57]
UCB-TT52CAR19: an umbilical cord blood derived, anti-CD19 CAR-T for B-cell malignancy	$CD62L^+$ donor UCB-T cells were engineered by lentiviral transduction. CRISPR Cas-9 was used to knock out the *TRAC* and *CD52* loci.	In vitro and in vivo models of $CD19^+$ B-cell malignancy	UCB-TT52CAR19 achieved effective cytotoxicity in vitro, comparable to peripheral blood derived CAR T cells and greater than untransduced control cells. They exhibited enhanced anti-tumour activity in vivo compared to untransduced controls and at a broadly similar level to peripheral blood derived CAR T cells.	[123]

Abbreviations:, AAV: Adeno-Associated Virus, AML: Acute Myeloid Leukaemia, B-ALL: B cell acute lymphoblastic leukaemia, BCMA: B cell maturation antigen, BPDCN: Blastic Plasmacytoid Dendritic Cell Neoplasm, chRDNA: CRISPR Hybrid RNA-DNA, CRISPR-Cas9: Clustered regularly interspaced short palindromic repeats-CRISPR-associated protein 9, CRS: Cytokine Release Syndrome, CTCL: Cutaneous T cell Lymphoma, GVHD: Graft-vs-Host Disease, HDR: Homology-directed repair, HIP: Hypoimmune, KO: Knockout, LBCL: Large B cell Lymphoma, LLT1: Lectin-like transcript 1, LTR: Long Terminal Repeat, NK: Natural Killer Cell, PBMC: Peripheral Blood Mononuclear Cells, PBS: Phosphate-buffered Saline, PDX: Patient Derived Xenograft, PTCL: Peripheral T cell Lymphoma, Ref: Reference, rhIL-7-hyFc: long-acting interleukin-7, RNP: ribonucleoprotein, r/r: relapsed or refractory, SB: Sleeping Beauty, TALEN: Transcription Activator-Like Effector Nucleases, T-ALL: T cell Acute Lymphoblastic Leukaemia, TCR: T cell Receptor, T-NHL: T cell non-Hodgkin's lymphoma, Treg: regulatory T cell, UCART: universal CAR-T cells

Supplementary Table S2. Preclinical studies using Non-Genome-Edited CAR cells.

Description	CAR production	Study design	Key Findings	Ref.
CNTY-101: an anti-CD19, iPSC-derived CAR-NK for B-cell Autoimmune Disease*	iPSCs were engineered using the CRISPR MAD-7 nuclease to knock out *B2M* and *CIITA* and to knock in the CD19 CAR, *HLA-E*, secreted IL-15 and EGFR safety switch. Cells were then differentiated into CAR-NK.	In vitro models of B cell autoimmune disease	CNTY-101 depleted B cells (including those from SLE patients) more effectively than autologous CAR-T. This was accompanied by reduced production of inflammatory cytokines (TNF-α + IFN-γ). When tested in an in vitro persistence assay, cells remained for longer than than iPSC-derived NK cells lacking edits, with further increase by IL-2 addition. CNTY-101 was protected from $CD8^+$ T cell activation and lysis, in contrast to iPSC-derived NK cells. In addition, there was negligible lysis of HLA-E expressing CNTY-101 by NK cells. Cetuximab effectively depleted CNTY-101, confirming utility of the EGFR safety switch.	[48]
NK-CAR12: an anti-CD19 CAR-NK for leukaemia /lymphoma	NK-92 cells (an immortalised NK cell line) was engineered by retroviral transduction.	In vitro and in vivo models of leukaemia/ lymphoma:	In vitro and in vivo functional studies confirmed that the optimised CAR candidate for expression in NK-92 cells contained a CD8α hinge, CD28 transmembrane domain and fused DAP10-2B4-CD3ζ endodomain.	[133]
^{Allo15}CAR33-NKT: an anti-CD33 CAR-NKT cell therapy for myeloid malignancy	Lentiviral transduction of haematopoietic stem cells to express an invariant NK TCR, CAR and secreted IL-15. Cells were then differentiated to NKT cells.	In vitro and in vivo models of myeloid malignancy	A high yield of CAR33-NKT was achieved using a clinically guided culture method. When evaluated in a PDX model, ^{Allo15}CAR33-NKT expanded over 200 fold and persisted for 50 days, without causing GVHD. By contrast, conventional CD33 CAR-T expanded 400 fold but caused severe GVHD. Efficacy of CAR33-NKT cells was demonstrated in three human AML xenograft models, and in models of antigen escape and high-risk AML.	[88]
CD19 CAR-UiNK: an embryonic stem cell (ESC) derived, anti-CD19 CAR-NK for B cell leukaemia /lymphoma	An ESC cell line was edited using Cas12iHiFi to knock out the *B2M* locus. PiggyBac vector transduction was performed to co-express the CAR and HLA-E (containing HLA-G signal peptide and B2M). Cells were then differentiated to NK cells.	In vitro and in vivo models of B cell malignancy	CD19 CAR-UiNK cells achieved enhanced cytotoxicity compared to differentiated NK control cells. When tested in a B-ALL xenograft model, they mediated stronger tumour killing leading to increased survival when compared to mice treated with differentiated control NK cells or PBS.	[92]
(12C) CD5 CAR-NK: an anti-CD5 CAR-NK for T cell malignancies	Healthy donor peripheral blood NK cells were engineered by lentiviral transduction to co-express a	In vitro and in vivo models of T cell malignancies	CAR expression resulted in fratricide of T cells, but not NK cells which lack CD5. CAR NK cells achieved enhanced cytotoxicity, in vivo tumour control and survival, when compared to nontransduced control	[111]

	nanobody derived CAR and secreted IL-15.		NK cells.	
CAR-NK with protease inhibitor: an anti-CD33 or CD70 CAR-NK for AML	Peripheral blood-derived NK cells were engineered by retroviral transduction	In vitro and in vivo models of AML	CAR-NK cells targeted against CD33 or CD70 had enhanced cytotoxicity against target cells lines compared to activated nontransduced NK cells. Pre-treatment of target cell lines and primary AML samples with protease inhibitors further enhanced cytotoxicity. When tested in vivo in xenograft models of AML, pre-treatment with bortezomib followed by CAR-NK infusion significantly enhanced tumour control and survival.	[119]
CAR-TIM3 NK-92: an anti-TIM3 CAR-NK for AML	CAR expression was achieved in NK-92 cells by lentiviral transduction	In vitro and in vivo models of AML	CAR NK-92 cells achieved enhanced cytotoxicity of TIM3^{+} AML cells compared to unmodified control NK-92 cells, but had little detrimental effect on haematopoietic stem cells. In vivo models showed that repeated CAR-TIM3 NK-92 infusions reduced tumour burden significantly compared to control NK-92 cells.	[156]
CD70-CAR NK: an anti-CD70 CAR-NK for B cell lymphoma	Umbilical cord blood-derived NK cells were engineered by lentiviral transduction to co-express IL-15 and an scFv-based CAR	In vitro and in vivo models of B cell lymphoma	CD70-CAR and CD19-CAR NK cells showed potent, selective cytotoxicity in against target tumour cells. CD70-CAR NK cells that expressed IL-15 achieved significant control of CD19 negative tumours, resulting in enhanced survival compared to CD19-CAR controls. This effect was potentiated by repeated infusion of CD70 CAR NK cells.	[112]
anti-CD19 CAR-NK: an anti-CD19 CAR-NK for leukaemia + lymphoma	Peripheral blood-derived NK cells were engineered by retroviral transduction, making comparison with CAR-T cells.	In vitro and in vivo models of lymphoma/ leukaemia	Both CAR-T + CAR-NK cells achieved significantly higher cytotoxicity in vitro than nontransduced control cells. However, cytotoxicity, IFN-γ production and in vivo efficacy against either a lymphoblastoid or leukaemic xenograft model were all greater by CAR-T cells than by CAR-NK cells.	[141]
BCMA-CD28-IL15 CAR-NK: an anti-BCMA CAR-NK for multiple myeloma	Peripheral blood-derived NK cells were engineered by lentiviral transduction to co-express a CAR and IL-15	In vitro and in vivo models of multiple myeloma	BCMA-CD28-IL15 CAR-NK cells demonstrated significantly increased cytotoxicity in vitro compared to non-IL15-producing BCMA CAR-NK cells or nontransduced NK cells. Incorporation of a CD28 hinge region was linked to enhanced cytotoxicity compared to an IgG1 hinge. In some models, inclusion of a CD28 intracellular domain was associated with better cytotoxicity compared to a 2B4 domain. When tested in vivo, CD28-containing CAR NK cells achieved significantly greater tumour control and survival than mock-transduced NK cells.	[113]
BCMA CAR-iNKT cells: an anti-BCMA CAR for multiple myeloma	iNKT cells from donor PBMCs were engineered by lentiviral transduction	In vitro and in vivo models of multiple myeloma. Therapy was tested +/- rhIL-7-hyFc (long-acting IL-7 analogue)	BCMA CAR iNKT cells achieved similar in vitro antitumour efficacy to BCMA CAR-T cells. When tested in vivo, BCMA CAR iNKT cells elicited a significant reduction in tumour burden and enhanced survival compared to CD19 CAR-iNKT control cells. Addition of rhIL-7-hyFc enhanced in vivo expansion and improved efficacy and survival.	[73]

NK-92- CAR.19-IL-15/IL-15Rα: an anti-CD19 CAR-NK for B cell leukaemia/ lymphoma	NK-92 cells were engineered by lentiviral transduction to co-express CAR and membrane bound IL-15 linked to IL-15Rα	In vitro and in vivo models of B cell leukaemia/ lymphoma	Cytotoxicity of NK-92 cells against B cell targets was enhanced by CAR and IL-15/ IL-15Rα expression. In repeated tumour stimulation models, CAR NK-92 cells that co-expressed 15/ IL-15Rα had lower increase in PD-1 and LAG-3 exhaustion marker expression. In vivo efficacy of NK-92-CAR.19-IL-15 /IL-15Rα was also better than controls that co-expressed soluble IL-15 or no cytokines.	[115]
anti-TCRVβ CAR-iNKT cells: an CAR-iNK therapy for T cell lymphoma targeted against clonotypic TCRVβ chain	iNKT cells from donor PBMCs were engineered by lentiviral transduction	In vitro and in vivo models of T cell lymphoma	CAR-iNKT cells directed against TCRVβ1, 2 or 9 efficiently killed target cells in vitro whereas cells re-targeted against TCRVβ11 could not be expanded due to fraticide. Addition of αGalCer, a glycolipid ligand of iNKT cells, enhanced their cytotoxicity unlike CAR-T cells which were unaffected. TCRVβ2-specific CAR-iNKT cells were cytotoxic against T cell lymphoma cells derived from patients and significantly reduced tumour burden in a T cell lymphoma xenograft model, when compared to CD19-specific CAR-iNKT or nontransduced control cells.	[157]
iDuo NK: an iPSC derived, anti-CD19 CAR-NK, for B-cell leukaemia and lymphoma	iPSCs were engineered by a nondisclosed method to co-express IL-15 linked to IL-15Rα and a high affinity noncleavable variant of CD16 (hnCD16).<br>CAR-NK cells were then derived by differentiation.	In vitro and in vivo models of B cell leukaemia/ lymphoma	iDuo NK cells exhibited robust cytotoxicity against cognate target cells, compared to nontransduced controls. The addition of rituximab facilitated killing by ADCC against CD19 negative target cells. The presence of IL-15 linked to IL-15Rα enhanced both target-specific and innate cytotoxicity. Moreover, iDuo NK cells preferentially targeted $CD19^+$ tumour over $CD19^+$ healthy cells. iDuo NK cells demonstrated in vivo efficacy in a number of models which was potentiated by rituximab.	[104]
CAR.CD123-NK: an anti-CD123 CAR-NK for paediatric AML	Peripheral blood-derived NK cells were engineered by retroviral transduction	In vitro and in vivo models of AML	CAR.CD123-NK cells achieved enhanced cytotoxicity against $CD123^+$ patient samples and an AML xenograft model. Expansion of the cells was demonstrated in a humanised mouse model of AML. Notably, CAR.CD123-NK cells caused a marginal depletion of healthy $CD123^+$ cells and did not target endothelial cells in contrast to conventional CAR.CD123-T cells, which caused severe bone marrow aplasia and endothelial destruction.	[158]
CD123CAR-δ1 T cell (DOTs): an anti-CD123 CAR-DOT for AML	Peripheral blood-derived DOT cells were engineered by retroviral transduction	In vitro and in vivo models of AML	CD123 CAR-DOT cells achieved comparable cytotoxicity to CD123CAR-T cells, both of which were greater than mock transduced control T cells or DOT cells. In an AML xenograft model, serial infusions of CD123CAR-DOT cells controlled tumour burden significantly more effectively than mock-transduced DOT cells. Efficacy of this approach was further boosted using exogenous IL-15.	[76]
CCR7-CD19 t-haNK: an anti-CD19 CAR-NK for lymphoma	NK-92 cells were electroporated to co-express a CD19 CAR, CCR7 receptor, CD16 and endoplasmic reticulum-retained IL-2	In vitro and in vivo models of lymphoma	t-haNK cells demonstrated cytotoxicity against $CD19^+$ cells and against $CD19^-$ cell lines in the presence of rituximab. When tested in vivo , incorporation of CCR7 was shown to improve tumour infiltration and disease control.	[140]

ARI3: an anti-CD19 UCB derived CAR-NK for NHL and multiple myeloma	Umbilical cord blood-derived NK cells were engineered by lentiviral transduction	In vitro and in vivo models of NHL and multiple myeloma	ARI3 CAR-NK cells achieved robust cytotoxicty against NHL cells only at high E:T ratios and in a manner that was boosted by exogenous IL-2. However, combination therapy with CAR-T cells potentiated overall efficacy, maintaining CAR-T cell fitness and migration to the site of disease without increased toxicity.	[124]
CD38CAR KHYG-1: an anti-CD38 CAR-NK for multiple myeloma	Retroviral transduction of the immortalised NK cell line, KHYG-1	In vitro and in vivo models of multiple myeloma	CD38CAR KHYG-1 selectively lysed myeloma cells in bone marrow samples. A medium affinity CAR proved significantly more effective than a low affinity CAR. Irradiated CD38CAR KHYG-1 cells maintained the ability to mediate tumour cell cytotoxicity and in vivo tumour control. Cytotoxicity was also shown against bone marrow cells derived from daratumumab refractory patients.	[127]
γδCAR-T: an anti-CD19 CAR-γδ for B-cell leukaemia	Retroviral transduction of zoledronic acid activated PBMCs	In vitro and in vivo models of B cell malignancies	γδ CAR-T and standard CAR-T cells mediated cytotoxicity against CD19-expressing targets while γδCAR-T + γδT cells had significant cytotoxicity against CD19 knock out cell lines which was enhanced by pre-exposure to zoledronic acid. γδCAR-T and standard CAR-T cells showed significant in vivo antitumour activity when compared to controls, although residual leukaemic burden was higher with γδCAR-T. Although they demonstrated limited persistence, efficacy of γδCAR-T could be boosted by re-dosing with zoledronic acid.	[103]
CAR.CD19-NK: an anti-CD19 CAR-NK for B cell precursor-ALL	Peripheral blood-derived NK cells were engineered by retroviral transduction	In vitro and in vivo models of B cell precursor-ALL	CAR.CD19-NK achieved superior cytotoxicity (including B cell precursor ALL target cells) and xenograft control compared to untransduced NK cells. However, they were ineffective against CD19 negative cells. Rituximab enhanced efficacy against $CD19^{+}CD20^{+}$ cell lines.	[159]
CD19-CAR-NKT: an anti-CD19 CAR-NK for lymphoma	NKT cells from healthy donor PBMCs were subjected to retroviral transduction	In vitro and in vivo models of lymphoma	CD19-CAR NKT demonstrated superior cytotoxicity compared to controls. Expansion of the cells in the additional presence of IL-21 (compared to IL-2 alone) increased cytotoxicity (especially of $CD62L^{+}$ cells), resulting in significantly increased survival of tumour-bearing mice. CAR-NKT treated mice did not develop GVHD or other toxicity.	[116]
iCasp9/CAR.19/IL-15 UCB-NK: an anti-CD19, UCB-derived, CAR-NK for B cell malignancies	Umbilical cord blood-derived NK cell were engineered by retroviral transduction to co-express CAR, IL-15 and inducible caspase 9 (iCasp9)	In vitro and in vivo models of B cell malignancy	Fully engineered NK cells achieved greater cytotoxicity against $CD19^{+}$ cell lines and primary CLL cells compared to untransduced NK cells, but killing was equivalent against non-target CD19 negative cell lines. In the case of autologous CLL patient cells, CAR transduction only marginally increased efficacy. Engineered NK cells controlled tumour burden and prolonged survival in xenograft bearing mice for significantly longer than untransduced NK cells or NK cells that expressed the CAR without IL-15. An increased dose of the engineered NK cells resulted in CRS but addition of the AP1903 dimeriser agent induced effective iCasp9-mediated clearance of the cells.	[98]

CD5-VLR or CD5-scFv-CAR: anti-CD5 CAR-NK for T-ALL	Lentiviral transduction of immortalised NK-92 cells,	In vitro and in vivo models of T-ALL	Comparison was made between an scFv and VLR-based CAR. VLR are found in jawless vertebrates and are analogous to immunoglobulin. In vitro, both CD5 CAR-NK constructs enabled significantly enhanced cytotoxicity, although this was greater for the scFv-based CAR. When tested in vivo, both CARs mediated tumour control although survival was significantly greater for the scFv compared to VLR-based CAR.	[134]
CIK/63.28.z: an anti-CD19 CAR-CIK for pre-B-ALL	Lentiviral transduction of cytokine induced killer (CIK) cells from healthy donor PBMCs	In vitro and in vivo models of pre-B-ALL	CAR-CIK cells mediated killing of $CD19^+$ cells and pre-B-ALL blasts. When tested in vivo, they achieved greater expansion and mediated improved disease control and enhanced survival in CD19 xenograft models, when compared to untransduced CIK control cells. However, GVHD was seen in some cases.	[85]
CS1-CAR NK-92 and CS1-CAR NK-L: anti-CS1 CAR-NK for MM	Lentiviral transduction of immortalised NK-92 or NK-L cell line	In vitro and in vivo models of multiple myeloma	Expression of the CS1-CAR enhanced in vitro cytotoxicity of both NK-92 and NK-L cells against $CS1^+$ targets, when compared to mock transduced controls. They also achieved higher levels of primary myeloma cell lysis compared to mock transduced controls, accompanied by enhanced release of IFN-γ. When evaluated in vivo, NK-92-CS1-CAR cells significantly supressed tumour burden and enhanced survival compared to mock transduced controls in mouse xenograft models.	[125]
mRNA-CD5-CAR-$\gamma\delta$T CD5 knock out: an anti-CD5 CAR-$\gamma\delta$ for T-ALL	$\gamma\delta$ cells from healthy donor PBMCs were engineered by CRISPR Cas-9 to knock out the *CD5* locus. CAR mRNA was delivered by electroporation.	In vitro and in vivo models of T-ALL	CD5 gene disruption effectively prevented fratricide of CD5 CAR-engineered $\gamma\delta$ T cells. The cells mediated specific lysis of CD5-expressing target cells in vitro, when compared to untransduced-$\gamma\delta$T CD5 knock out cells. CAR delivery by mRNA electroporation enabled similar antitumour efficacy to lentiviral transduction. Cells proved safe and effective in two xenograft models although effects were transient, requiring repeated administration for maximum efficacy.	[93]
CAR NK-92/63.z: an anti-CD19 CAR-NK for B-cell leukaemia/lymphoma	Lentiviral transduction of immortalised NK-92 cells	In vitro and in vivo models of B cell leukaemia/lymphoma	$CD19^+$ cells (including primary pre-B-ALL cells) were lysed by NK-92/63.z (first generation), NK-92/63.28.z (second generation) and NK-92/137.z (second generation) cells when compared to untransduced controls. NK-92/63.z cells significantly delayed lymphoma growth without toxicity in a xenograft model, when compared to mice treated with untransduced NK-92 cells or PBS.	[160]
FLT3 CAR_sIL-15 NK: an anti-FTL3 CAR-NK for AML	Lentiviral transduction of NK-92 or umbilical cord blood-derived NK cells to co-express soluble (s)IL-15 and a FLT3-specific CAR	In vitro and in vivo models of AML	FLT3 CAR NK-92 cells showed enhanced in vitro and in vivo efficacy accompanied by significantly prolonged mouse survival compared to mock transduced or untransduced NK-92 cells. Umbilical cord blood derived FLT3 CAR-NK cells also significantly delayed tumour progression in a xenograft model, when compared to control cells. FLT3 CAR_sIL-15 NK cells significantly enhanced in vitro efficacy compared to FTL3 CAR-NK (without IL-15 secretion) or NK cells that produced sIL-15 without a CAR . When cryopreserved, cells exhibited comparable	[114]

			function to fresh cells in vitro and in vivo. Furthermore, they exhibited no evidence of toxicity to healthy CD34⁺ cells in vivo.	
CAR⁺CD45RA negative cells: an anti-CD19 CAR-T for leukaemia	Lentiviral transduction of healthy donor derived CD45RA negative cells	In vitro and in vivo models of leukaemia	In vitro, MLL-rearranged CD19 cells were selectively lysed by CAR⁺CD45RA⁻ cells. Additionally, cells resistant to NK cytotoxicity were susceptible to lysis by CAR⁺CD45RA negative cells. CD45RA negative cells showed significantly less proliferation and IFN-γ production in a mixed leucocyte reaction when compared to CD45RA⁺ cells, suggesting low level alloreactivity. CAR⁺CD45RA negative cells also demonstrated enhanced efficacy in xenograft-bearing mice compared to controls. Furthermore, these cells did not induce GVHD in contrast to CD45RA⁺ cells (irrespective of CAR expression).	[87]
iDuo-MM: an anti-BCMA CAR-iPSC-derived NK for multiple myeloma	iPSC engineered using an AsCpF1 nuclease containing RNP to co-express IL-15 linked to IL-15Rα and hnCD16 via targeted integration of both genes into the *CD38* locus. The CAR was delivered by lentiviral transduction and cells were then differentiated to NK cells	In vitro and in vivo models of multiple myeloma	iDuo-MM mediated effective cytotoxicity alone, or in combination with daratumumab, while untransduced iPSC-derived NK were only able to lyse target cells with daratumumab. Activity was also demonstrated using primary patient samples and in xenograft models in which greatest activity was seen with the triple combination of iDuo-MM, daratumumab and gamma secretase inhibition.	[105]
OR-NOT CAR-NK: a bivalent CAR-NK with anti-FTL3/CD33 CAR and endomucin inhibitory CAR for AML	NK cells from healthy donor PBMCs were engineered by retroviral transduction to express a tricistronic construct encoding an anti-FLT3/anti-CD33 bivalent activating (a)CAR, an anti- endomucin inhibitory (i)CAR and a "modified form" of IL-15.	In vitro and in vivo models of AML	Endomucin is uniquely expressed by healthy haematopoietic stem cells accounting for why it was the target of the iCAR. Accordingly, this created a functional NOT gate system that mediated AML cell killing but did not attack healthy haematopoietic stem cells. A number of these systems were tested in vivo. First, OR (FTL3 OR CD33) CAR-NK cells showed improved tumour suppression and significantly increased mouse survival compared to controls. Building on this, OR-NOT gated CAR-NK cells (with IL-15 support) also achieved significant disease control, but mitigated the killing of healthy cells compared to OR only NK-cells.	[106]
NK-92/4G8.28.z: an anti-FLT3 CAR-NK for B-ALL	Lentiviral transduction of NK-92 cells to co-express CAR and iCasp9.	In vitro and in vivo models of B-ALL	FLT3 CAR NK-92 cells achieved enhanced lysis of target lines and B-ALL blasts compared to non-signalling and untransduced controls. No off-target cytotoxicity towards healthy CD34⁺ cells was observed. When tested in vivo, mice bearing leukaemic xenografts had significantly delayed tumour growth compared to controls. Cytotoxic activity of the cells was abrogated upon addition of the iCasp9 dimeriser agent, AP20187.	[99]
B2M$^{-/-}$CIITA$^{-/-}$CD54$^{-/-}$CD58$^{-/-}$ CAR NK: an anti-CD19, iPSC-derived CAR-NK for B-cell malignancies	iPSC cells were edited using CRISPR Cas-9 to knock out the *B2M* and *CIITA* loci alone, or in	In vitro and in vivo models of B cell malignancy	All engineered iPSC-derived CAR NK cells showed comparable tumour cytolytic activity to peripheral blood-derived NK cells. When tested in vivo, B2M$^{-/-}$CIITA$^{-/-}$CD54$^{-/-}$CD58$^{-/-}$ iPSC-derived CAR NK showed	[90]

	addition to the *CD54* and *CD58* loci. Next, cells were engineered using an AsCpF1 nuclease containing RNP to achieve safe harbour targeting of CAR, IL-15/IL-15Rα) and hnCD16. Cells were then differentiated to generate CAR-NK.		significantly enhanced tumour suppression and survival compared to untreated tumour alone. Moreover, while the $B2M^{-/-}CIITA^{-/-}$ iPSC CAR-NK were significantly depleted by NK cells, multi-edited $B2M^{-/-}CIITA^{-/-}CD54^{-/-}$ $CD58^{-/-}$ iPSC CAR-NK cells resisted rejection. HLA sufficient ($B2M^{+}$ $CIITA^{+}$) $CD54^{-/-}CD58^{-/-}$ iPSC-NK cells showed effective and similar in vitro and in vivo cytotoxicity, but had significantly enhanced persistence in assays of NK cell mediated rejection, highlighting the need for adhesion ligands in this process.	
CD117 CAR iNKT: an anti-CD117 CAR-iNK for AML	iNKT cells were generated from PBMCs and engineered by retroviral transduction.	In vitro and in vivo models of AML	CD117 CAR iNKT cytotoxicity was similar to that of CD117 CAR T cells, with both superior to untransduced controls. CD117 CAR iNKT cells significantly increased survival in an AML xenograft model compared to untransduced and PBS controls.	[161]
upCAR-NK: an anti-CD19 iPSC-derived CAR-NK for B cell malignancy	iPSCs were edited using CRISPR Cas-9 to knock out *B2M*, *CIITA*, *TRA*, *PDCD1* and *CTLA4* loci. Plasmid vector transfection was used to deliver a CD19-specific CAR and CD24 (don't eat me signal). Cells were then differentiated to generate CAR-NK.	In vitro and in vivo models of B-cell malignancy	Engineered iPSC-derived CAR NK cells achieved significantly higher cytotoxicity against Nalm-6 and Raji B cell targets, accompanied by elevated production of IFN-γ compared to controls including iPSC-derived NK cells and peripheral blood derived NK cells. In vivo efficacy of the cells was also demonstrated in xenograft-bearing mice.	[91]
33BBz γδ CAR T: an anti-CD33 CAR-γδ for AML	Healthy donor γδ T cells were engineered by retroviral transduction	In vitro and in vivo models of AML	CAR γδ cells achieved sgnificantly enhanced in vitro cytotoxicity compared to untransduced controls and αβ CAR-T. In vivo, they resulted in a significant reduction of circulating AML cells compared to untransduced γδ T cells and non-treated mice, leading to prolonged survival. Moreover, cytokine production by these cells was low, suggesting that they may incur a lowered risk of CRS.	[96]
CAR4-Double Negative T cells (DNT): an anti-CD4 CAR-DNT for T-ALL and peripheral T cell lymphoma (PTCL)	DNTs from healthy donor PBMCs were engineered by retroviral transduction to express a CD4-targeted CAR (CAR4).	In vitro and in vivo models of T-ALL and PTCL	CAR4 DNT cells achieved significantly enhanced lysis of both target lines and primary blasts in vitro. Treatment of mice bearing T-ALL xenografts resulted in tumour infiltration by the cells and prolonged survival compared to controls. Similar trends were demonstrated in mouse models of PTCL. Culture of the cells in the presence of a PI3Kδ inhibitor, idelalisib led to higher levels of DNT cells, lower tumour cell count and significantly enhanced survival.	[86]
CD70 CAR NK: an anti-CD70 CAR-NK for multiple myeloma	NK cells were engineered using an undisclosed method.	In vitro and in vivo models of multiple myeloma	CD70 CAR NK cells elicited enhanced target-dependent cytotoxicity against myeloma cells, including those lacking BCMA expression, when compared to untransduced controls. When evaluated in vivo, CD70 CAR NK achieved greater suppression of myeloma tumour burden, when compared to untransduced NK cells.	[162]

GD2-CARrejTs (rejuvenated): an anti-GD2 CAR-EBV-CTL for extranodal NK/ T cell lymphoma (ENKL)	iPSCs used to generate LMP2rejTs were engineered using lentiviral transduction.	In vitro and in vivo models of ENKL	When tested in a ^{51}Cr release assay, GD2-CARrejTs had significantly greater cytotoxicity against a target cell line than GD2-specific CAR-T cells. Mice treated w/ GD2-CARrejTs showed significantly greater tumour suppression compared to untreated control mice.	[84]
CD7 CAR-iNKT: an anti-CD7 CAR-iNK for T-ALL + AML	iNKT cells were engineered by lentiviral transduction,	In vitro and in vivo models of T-ALL + AML	About 44% of iNKT cells are CD7 negative obviating the need for genome editing of CD7. CD7 CAR-iNKT cells killed tumour cells in vitro at significantly greater levels than control CD19 CAR-iNKT or mock transduced iNKT cells. They also significantly supressed T-ALL and AML xenograft growth leading to survival prolongation compared to control mice.	[163]
FT555: an anti-GPR5CD iPSC-derived CAR-iNK for multiple myeloma	iPSCs were engineered using a nondisclosed method to knock out *CD38* and to co-express hnCD16 and IL-15R linked to IL-15Rα). Cells were then differentiated to generate CAR-iNK.	In vitro and in vivo models of multiple myeloma	FT555 demonstrated specific cytotoxicity against GPR5CD^{+} target cells and derived tumours, as well as persistent antitumour activity in restimulation assays. Addition of daratumumab further enhanced cytotoxicity and in vivo tumour control.	[94]

Abbreviations: ADCC: Antibody-Dependent Cellular Cytotoxicity, AML: Acute Myeloid Leukaemia, B-ALL: B-cell Acute Lymphoblastic Leukaemia, BCMA: B-cell maturation antigen, CIK: Cytokine-Induced Killer cell, CLL: Chronic Lymphocytic Leukaemia, CRISPR-Cas9: Clustered regularly interspaced short palindromic repeats-CRISPR-associated protein 9, CRS: Cytokine Release Syndrome, DOT: Delta One T-cell, DNT: Double Negative T-cell, EBV-CTL: Epstein Barr Virus Specific Cytotoxic T-Lymphocytes, EGFR: Epidermal growth factor receptor, ENKL: Extranodal NK/T-Cell Lymphoma, ESC: Embryonic Stem Cell, E:T: effector to target, γδ: Gamma-delta, GVHD: Graft-vs-Host Disease, hnCD16: High-affinity, non-cleavable CD16, iCasp9: iCaspase-9, iCAR: inhibitory chimeric antigen receptor, IFNγ: Interferon-gamma, iNK: Invariant Natural Killer, iPSCs: Induced Pluripotent Stem Cells, NK: Natural Killer, NKT: Natural Killer T cell, PBMC: Peripheral Blood Mononuclear Cell, PBS: Phosphate Buffered Solution, PDX: Patient Derived Xenograft, PI3K: Phosphatidylinositol 3'kinase, PTCL: Peripheral T-cell Lymphoma, rhIL-7-hyFc: long-acting interleukin-7, RNP: ribonucleoprotein, scFV: Single-chain Variable Fragment, , SLE: Systemic Lupus Erythematosus, T-ALL: T-cell Acute Lymphoblastic Leukaemia, TCR: T-cell Receptor, TIM3: T cell immunoglobulin and mucin domain containing 3, TNFα: Tumour Necrosis Factor-alpha, VLR: Variable Lymphocyte Receptor.

* Although only containing in vitro data, this study focusing on autoimmune application of 'CNTY-101' has been included since this product has already been applied in a clinical study [49] and it provides very useful insight for the autoimmune review focus. Furthermore, the reference used was a Poster Presentation, with the same title, as opposed to the Abstract linked on EMBASE, since it had more graphical data and evidenced-based results [48].
